# Supplementary material for: Investigations into the Structure/Antibacterial Activity Relationships of Cyclam and Cyclen Derivatives
Source: Antibiotics (Basel). 2019 Nov 14;8(4):224. doi: 10.3390/antibiotics8040224 (PMC6963676; doi:10.3390/antibiotics8040224)
Supplement: Supplementary file 1 [file antibiotics-08-00224-s001.pdf]

Investigations on Structure/Antibacterial Activity Relationships of Cyclam and Cyclen Derivatives

Luis G. Alves[1,*], João F. Portel[2,3], Sílvia A. Sousa[2], Olga Ferreira[4,5], Stephanie Almada[1,4], Elisabete R. Silva[4,5], Ana M. Martins[3], Jorge H. Leitão[2,*]

1 Centro de Química Estrutural, Associação do Instituto Superior Técnico para a Investigação e Desenvolvimento, Av. Rovisco Pais 1, 1049-003 Lisboa, Portugal
2 IBB-Instituto de Bioengenharia e Biociências, Instituto Superior Técnico, Universidade de Lisboa, Av. Rovisco Pais 1, 1049-001 Lisboa, Portugal
3 Centro de Química Estrutural, Instituto Superior Técnico, Universidade de Lisboa, Av. Rovisco Pais 1, 1049-001 Lisboa, Portugal
4 BioISI - Biosystems & Integrative Sciences Institute, Faculdade de Ciências da Universidade de Lisboa, Campo Grande, 1749-016 Lisboa, Portugal
5 CERENA - Centro de Recursos Naturais e Ambiente, Instituto Superior Técnico, Universidade de Lisboa, Av. Rovisco Pais 1, 1049-001 Lisboa, Portugal

* Correspondence: luis.g.alves@tecnico.ulisboa.pt (L.G.A), jorgeleitão@tecnico.ulisboa.pt (J.H.L)

Supporting Information

Table S1. Hydrogen bond distances (Å) and angles (º) in compound **14**.

	D-H···A	d(D-H)	d(H···A)	d(D···A)	(DĤA)	Symmetry Operation
**14a**	N(2)-H(1N)···O(1)	0.90(3)	2.08(4)	2.869(4)	146(3)	---
	N(2)-H(1N)···N(2)	0.98(4)	1.71(4)	2.675(4)	164(4)	-x, 2-y, -z
	O(3)-H(3O)···O(1)	0.96(5)	1.57(5)	2.528(3)	177(5)	---
**14b**	N(4)-H(3N)···O(6)	0.97(3)	1.90(4)	2.705(4)	139(3)	x, 1+y, z
	N(4)-H(3N)···N(3)*	0.97(3)	2.40(3)	3.004(4)	120(3)	1-x, 2-y,1-z
	N(4)-H(4N)···O(5)	1.04(5)	1.80(5)	2.770(4)	153(4)	1-x, 1-y, 1-z
	O(7)-H(7O)···O(5)	0.91(6)	1.65(6)	2.553(3)	176(7)	-x, 1-y, 1-z

*Intramolecular hydrogen bond

Table S2. Crystal data and structure refinement for compounds **8** and **14**.

	**8**	**14**
Empirical formula	C26 H40 N4	C36 H54 F6 N4 O8
Formula weight	408.62	784.83
Temperature (K)	150(2)	150(2)
Wavelength (Å)	0.71073	0.71073
Crystal system	Monoclinic	Triclinic
Space group	C2/c	P-1
Unit Cell Dimensions:		
a (Å)	20.719(2)	9.578(1)
b (Å)	9.2432(8)	10.528(1)
c (Å)	14.962(1)	20.376(2)
α (º)	90	81.323(5)
β (º)	121.894(4)	82.311(6)
γ (º)	90	79.096(5)
Volume (Å^3)	2432.8(4)	1982.7(3)
Z	4	2
Calculated density (g m^{-3})	1.116	1.315
Absorption coefficient (mm^{-1})	0.066	0.110
F (000)	896	832
Crystal size (mm)	0.16 x 0.16 x 0.18	0.12 x 0.14 x 0.14
Theta range for data collection (º)	3.207 – 27.182	2.346 – 26.534
Limiting indices	$-26\leq h\leq 26$, $-11\leq k\leq 8$, $-14\leq l\leq 19$	$-11\leq h\leq 12$, $-13\leq k\leq 13$, $-25\leq l\leq 25$
Reflections collected/unique [R_{int}]	8410/2683 [0.0489]	17572/8095 [0.0578]
Completeness to θ (%)	99.5 (θ = 25.242)	99.1 (θ = 25.242)
Refinement method	Full-matrix least squares on F^2	Full-matrix least squares on F^2
Data/restraints/parameters	2683/0/141	8095/0/515
Goodness-of-fit on F^2	1.003	0.939
Final *R* indices [$I>2\sigma(I)$][1]	R_1 = 0.0528, wR_2 = 0.1154	R_1 = 0.0649, wR_2 = 0.1543
R indices (all data)[1]	R_1 = 0.1066, wR_2 = 0.1293	R_1 = 0.1438, wR_2 = 0.1798
Absorption correction	Multi-scan	Multi-scan
Largest diff. peak/hole (e Å^{-3})	0.213 and -0.189	0.774 and -0.303

[1] $R_1 = \Sigma ||F_0| - |F_c|| / \Sigma |F_0|$; $wR_2 = \{\Sigma[w(F^2_0 - F^2_c)^2]/ \Sigma[w(F^2_0)^2]\}^{1/2}$

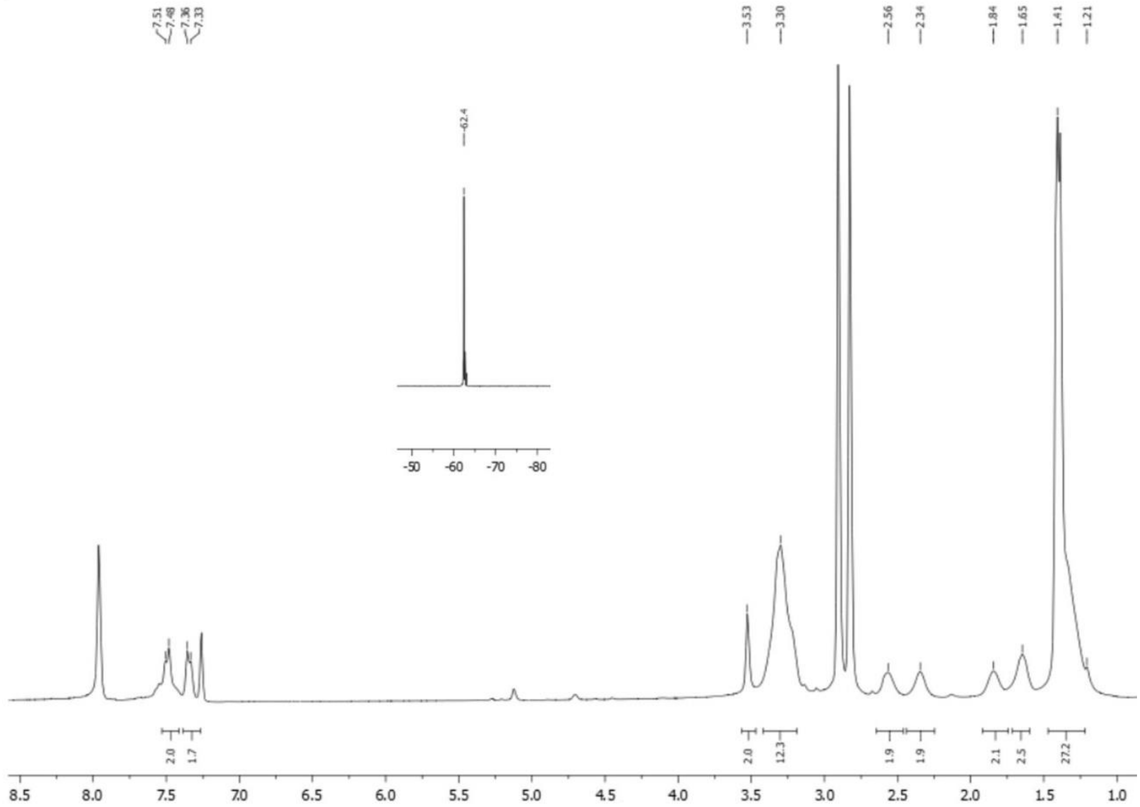

Figure S1A. ^{1}H and ^{19}F NMR spectra of compound **3** in $CDCl_3$.

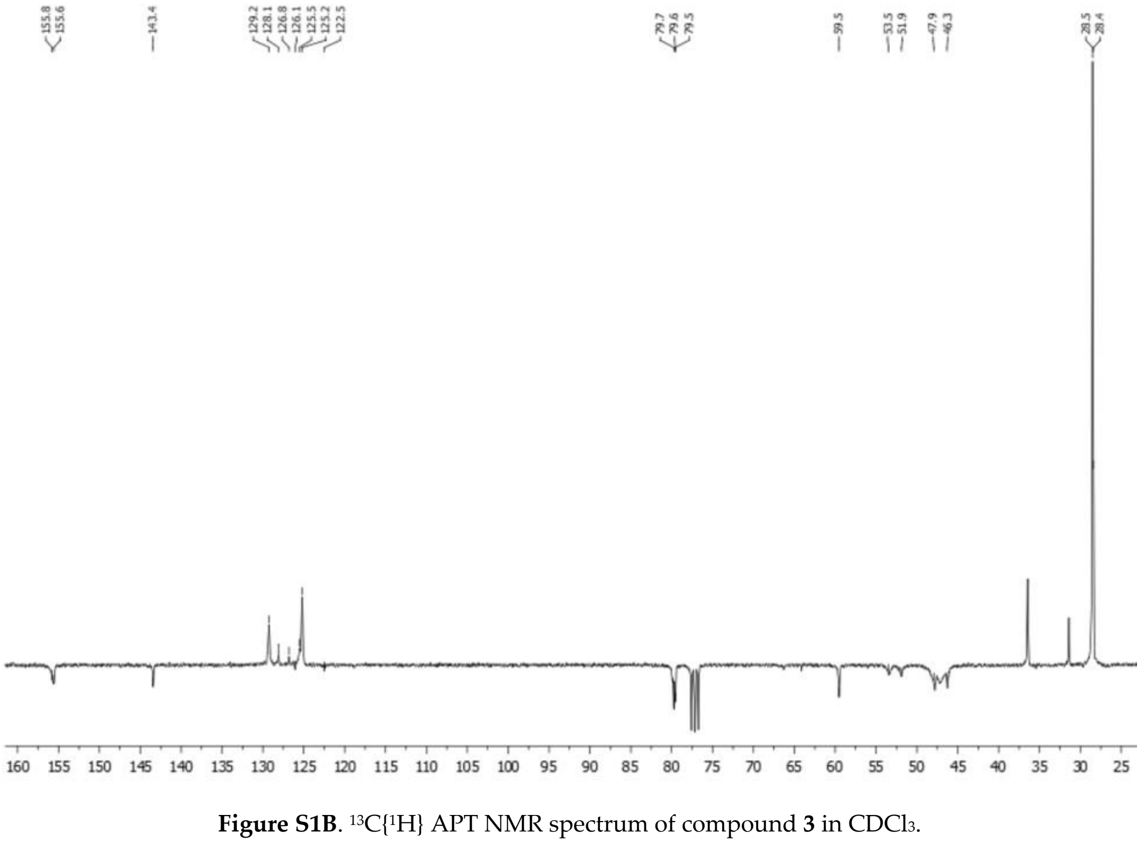

Figure S1B. $^{13}C\{^{1}H\}$ APT NMR spectrum of compound **3** in $CDCl_3$.

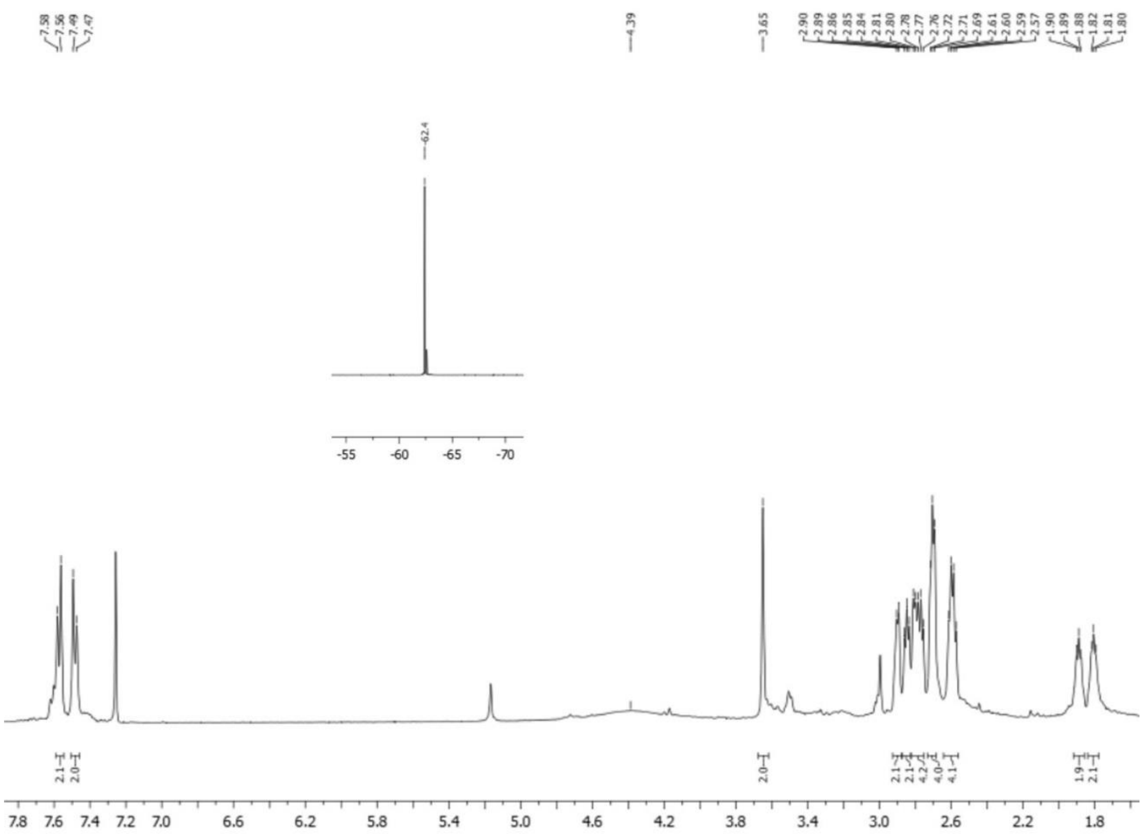

Figure S2A. ^{1}H and ^{19}F NMR spectra of compound **4** in $CDCl_3$.

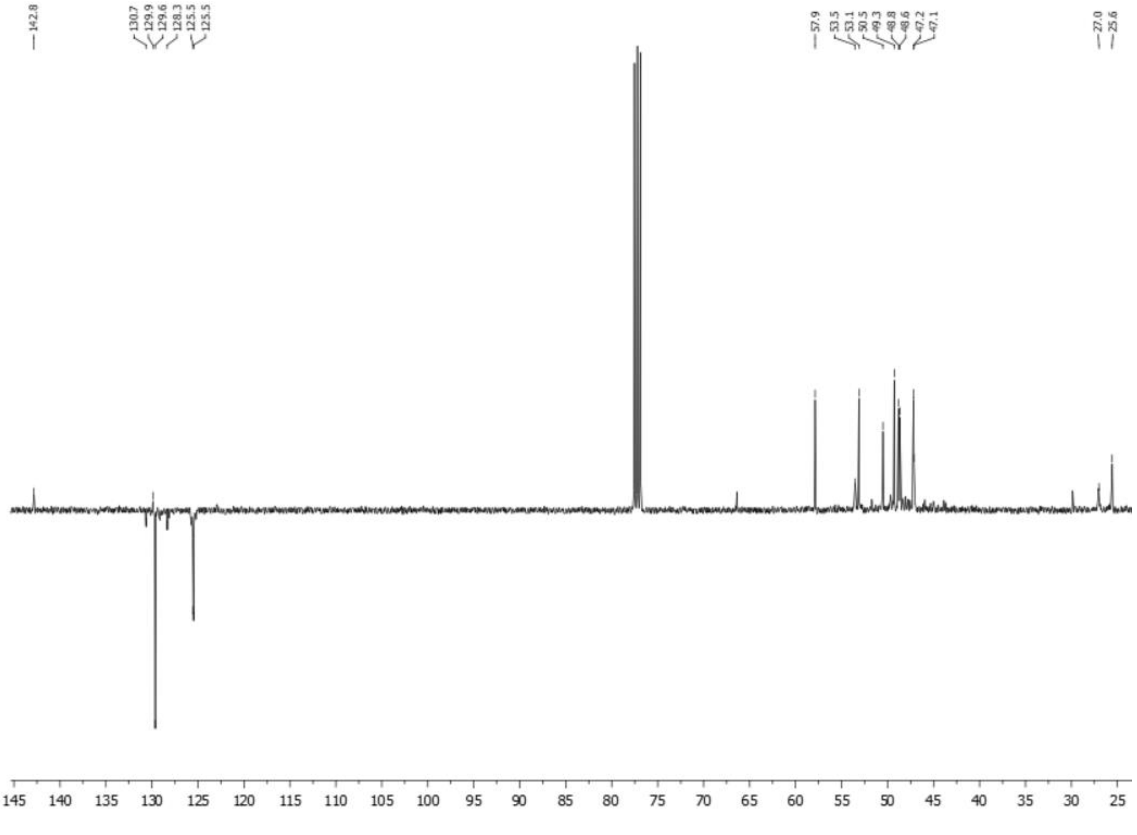

Figure S2B. $^{13}C\{^{1}H\}$ APT NMR spectrum of compound **4** in $CDCl_3$.

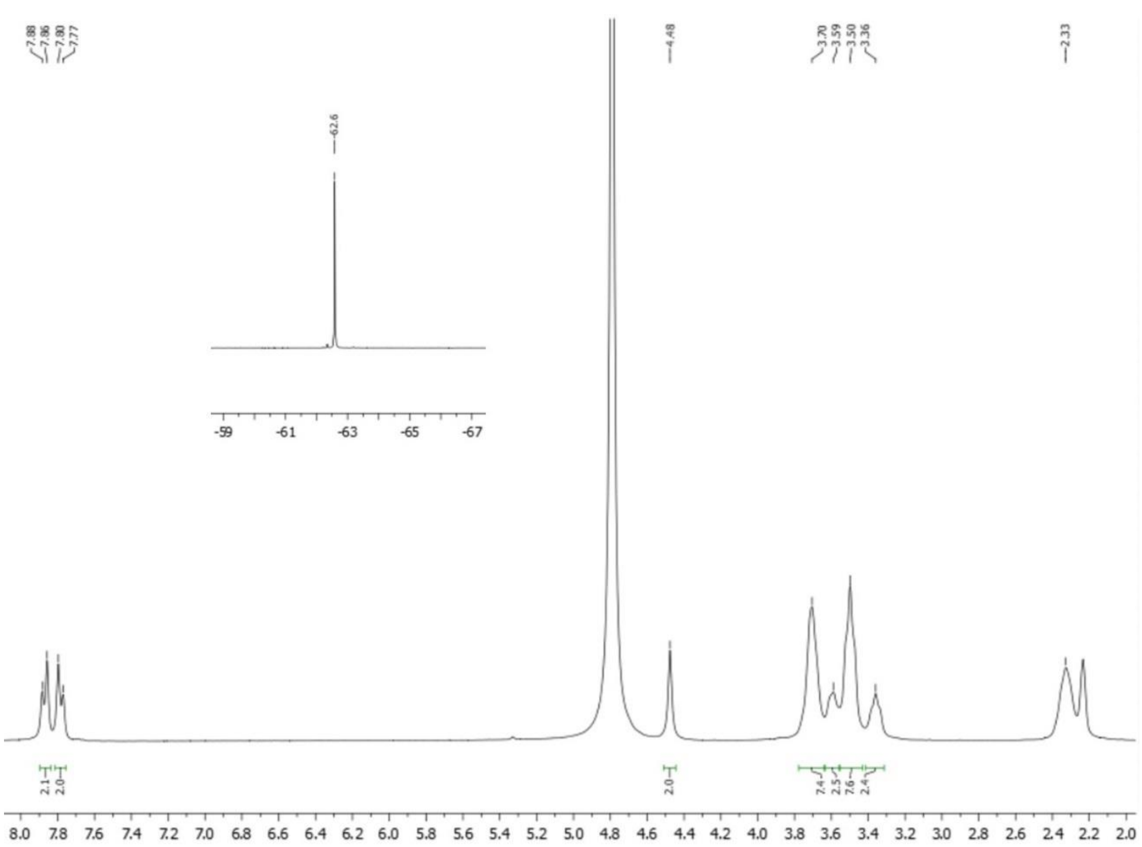

Figure S3A. ^{1}H NMR spectrum of compound **5** in $D_2O/(CD_3)_2CO$.

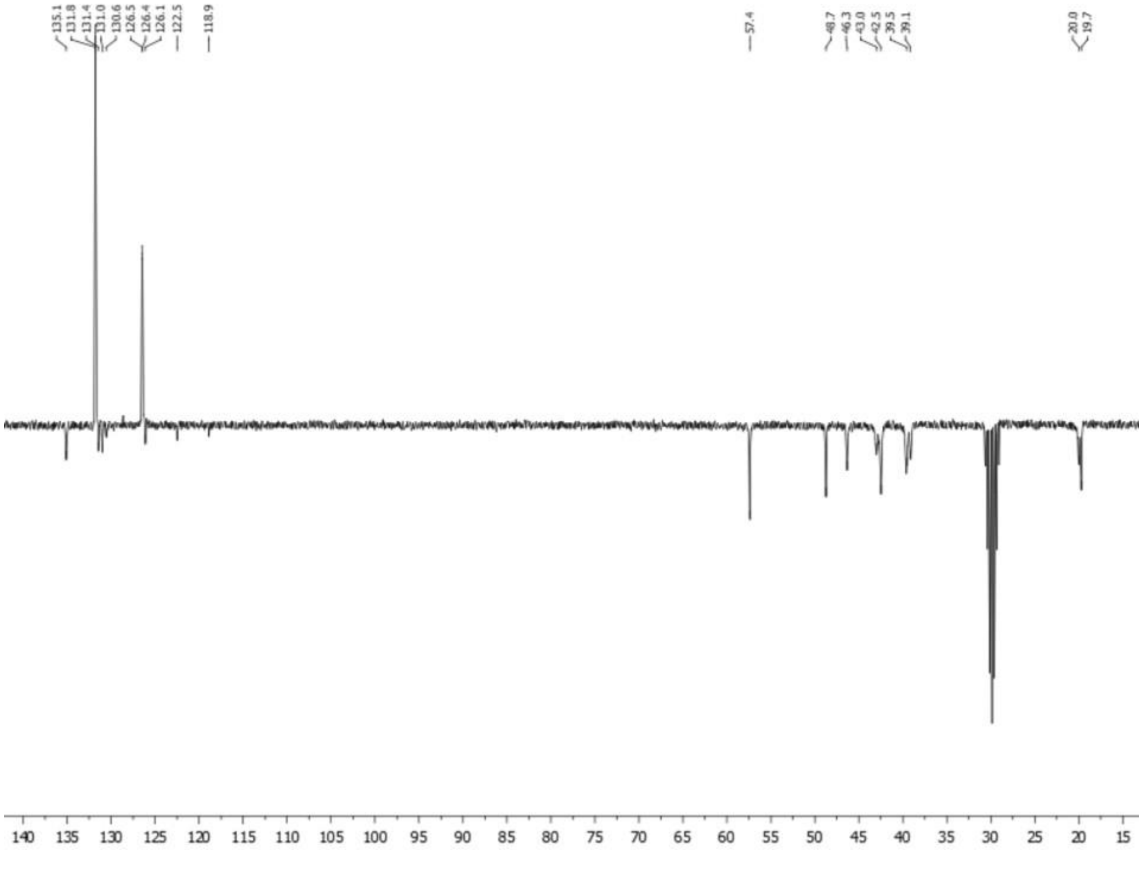

Figure S3B. $^{13}C\{^1H\}$ APT NMR spectrum of compound **5** in $D_2O/(CD_3)_2CO$.

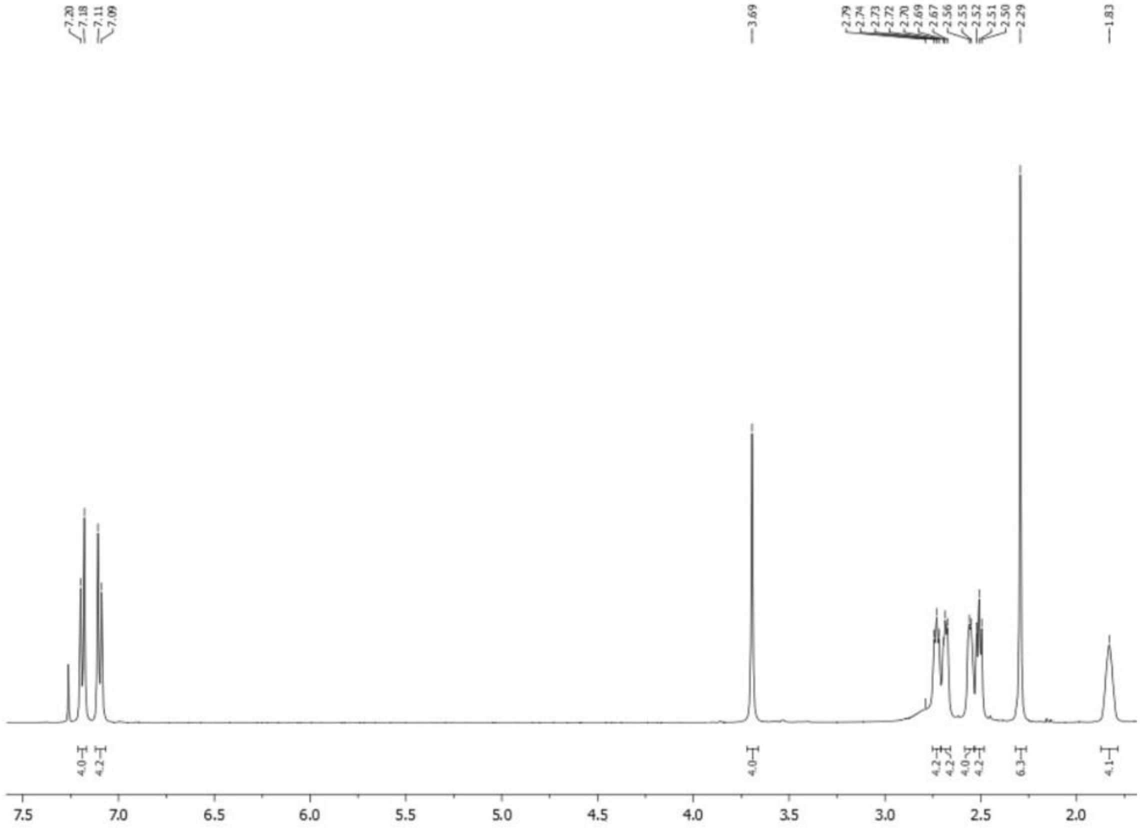

Figure S4A. ^{1}H NMR spectrum of compound **8** in $CDCl_3$.

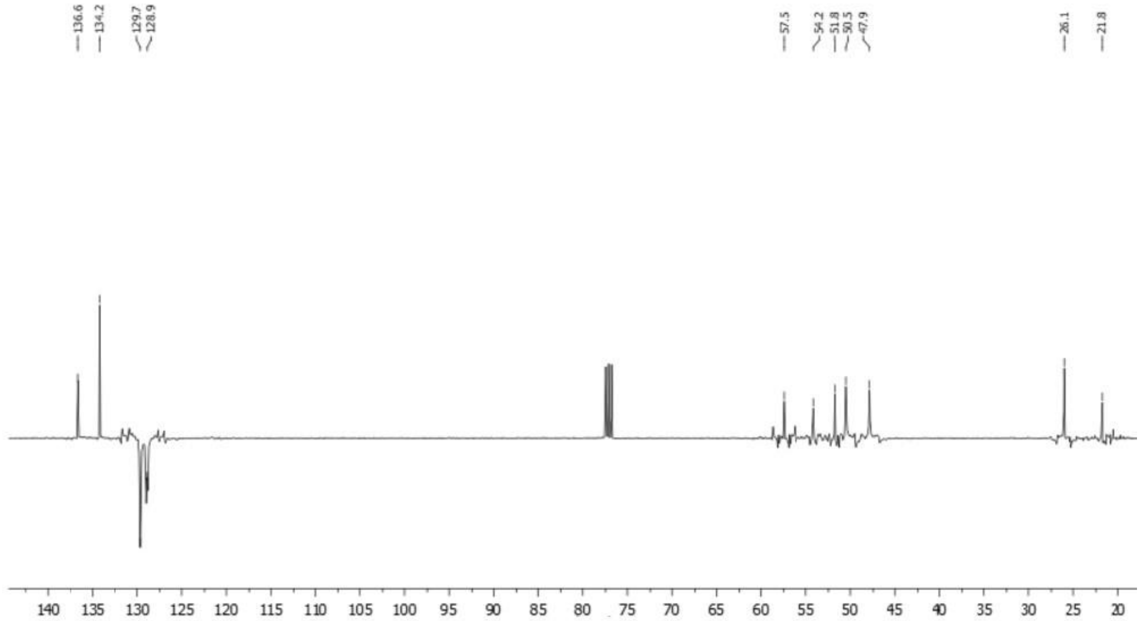

Figure S4B. $^{13}C\{^{1}H\}$ APT NMR spectrum of compound **8** in $CDCl_3$.

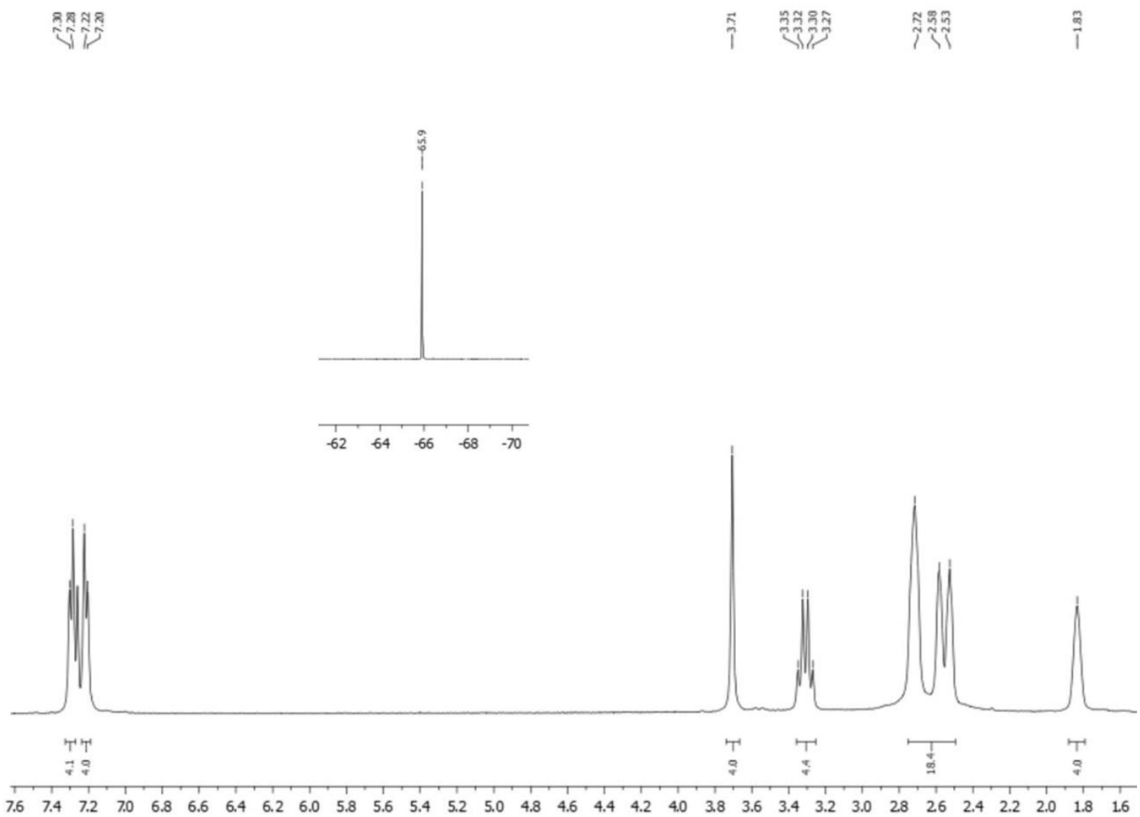

Figure S5A. ^{1}H NMR spectrum of compound **9** in $CDCl_3$.

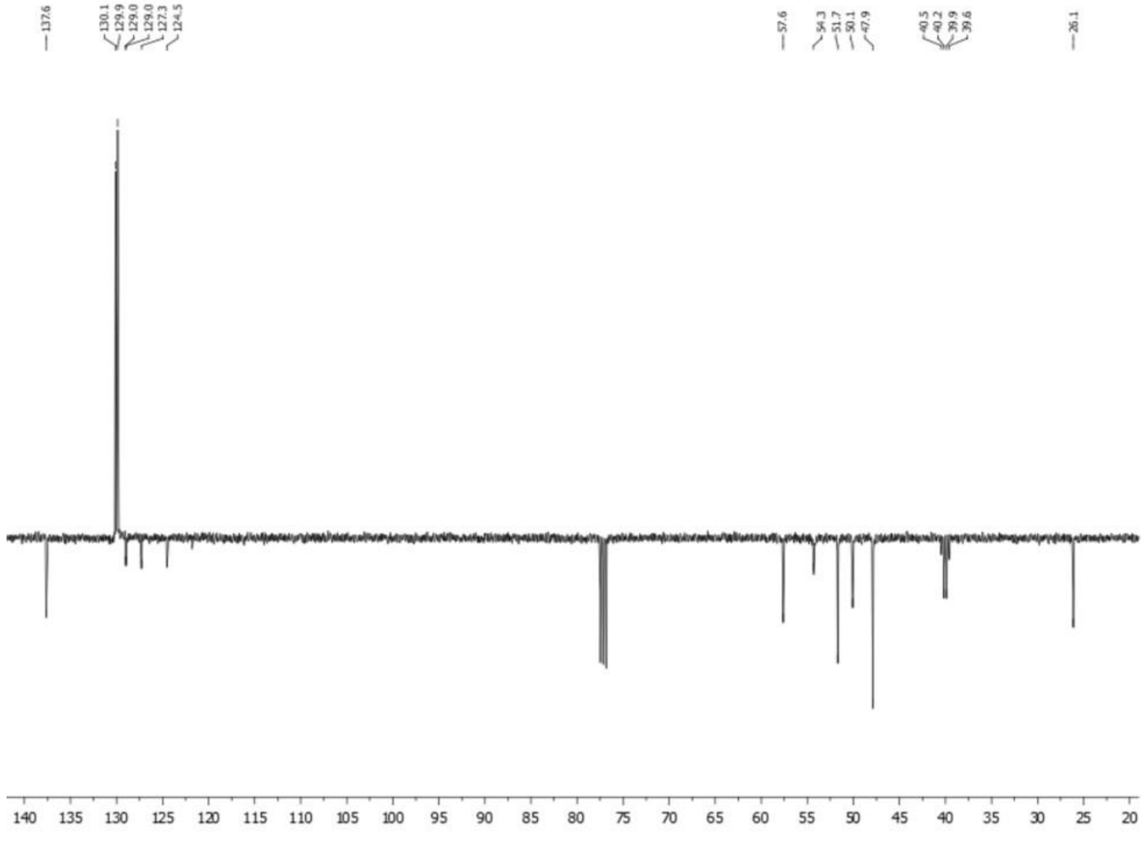

Figure S5B. $^{13}C\{^1H\}$ APT NMR spectrum of compound **9** in $CDCl_3$.

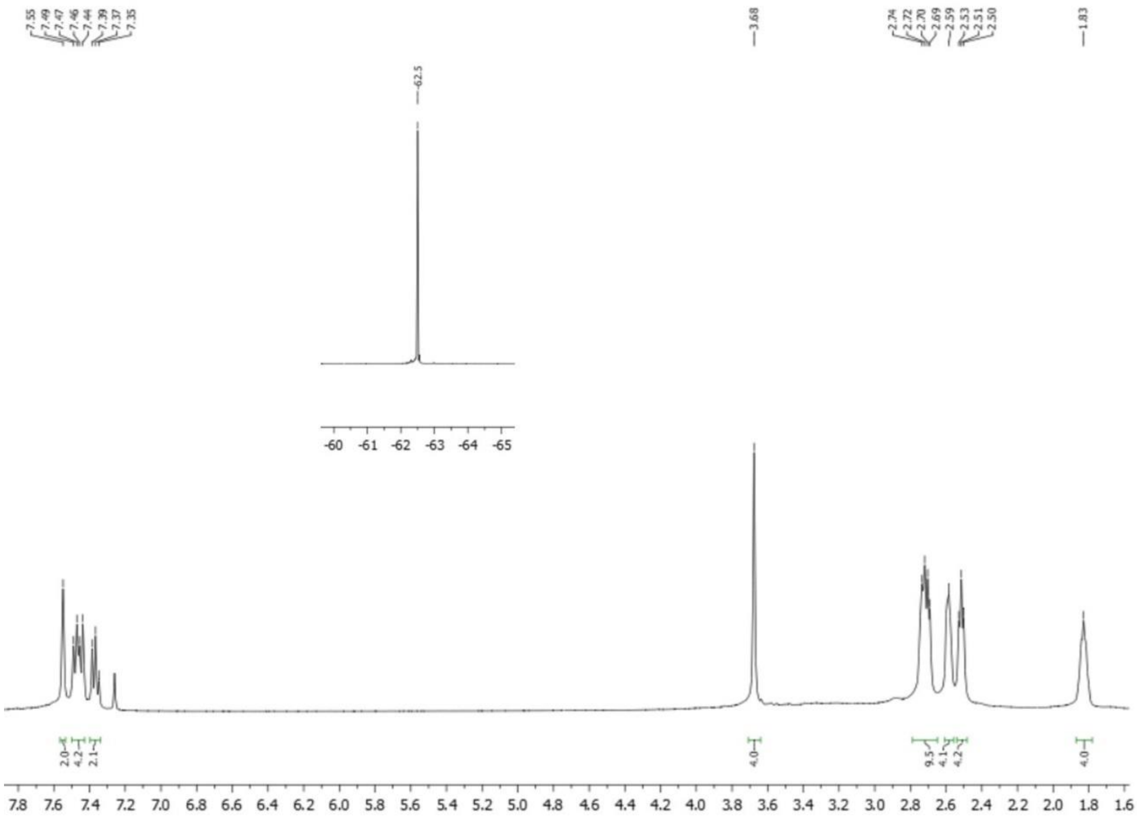

Figure S6A. ^{1}H NMR spectrum of compound **10** in $CDCl_3$.

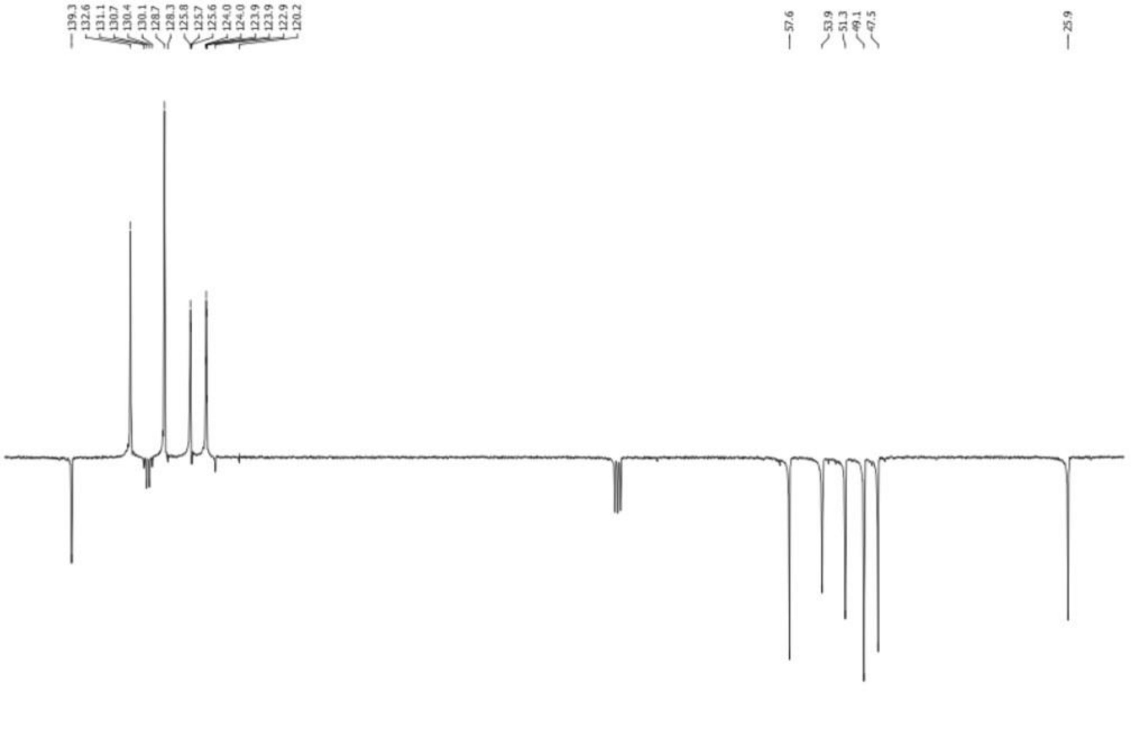

Figure S6B. $^{13}C\{^1H\}$ APT NMR spectrum of compound **10** in $CDCl_3$.

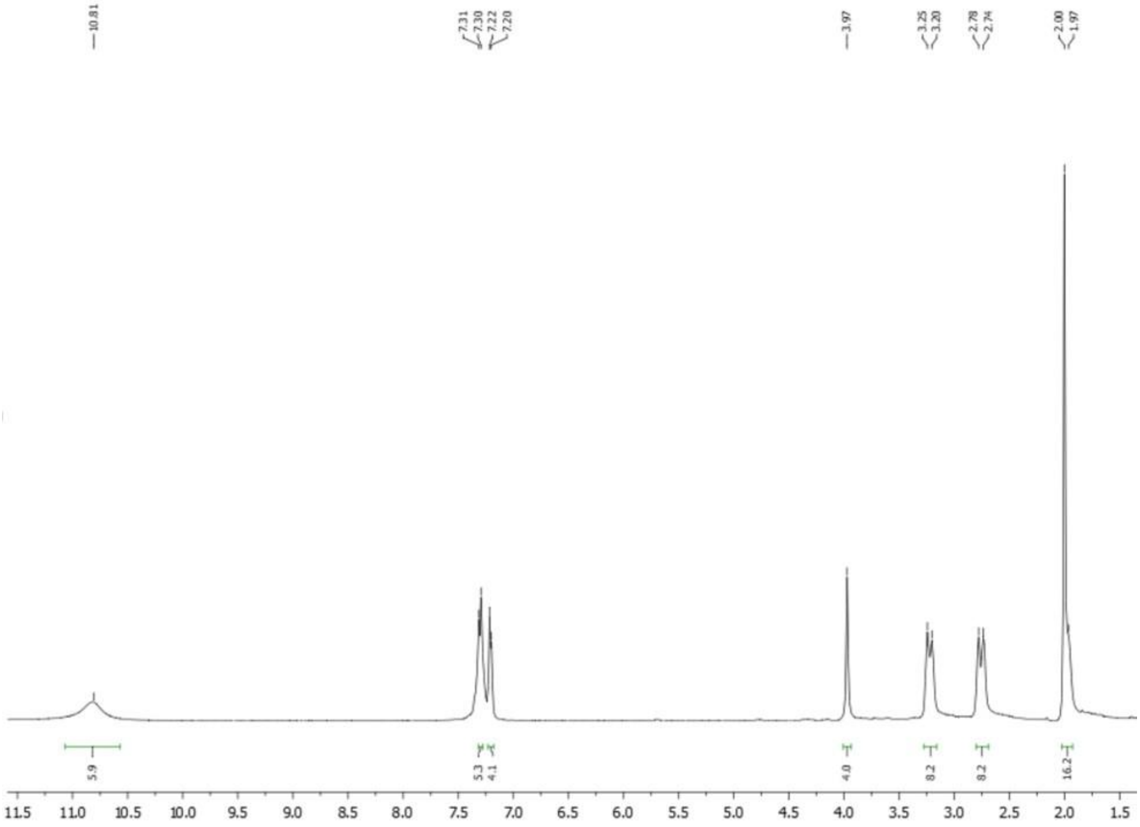

Figure S7A. ^{1}H NMR spectrum of compound **11** in $CDCl_3$.

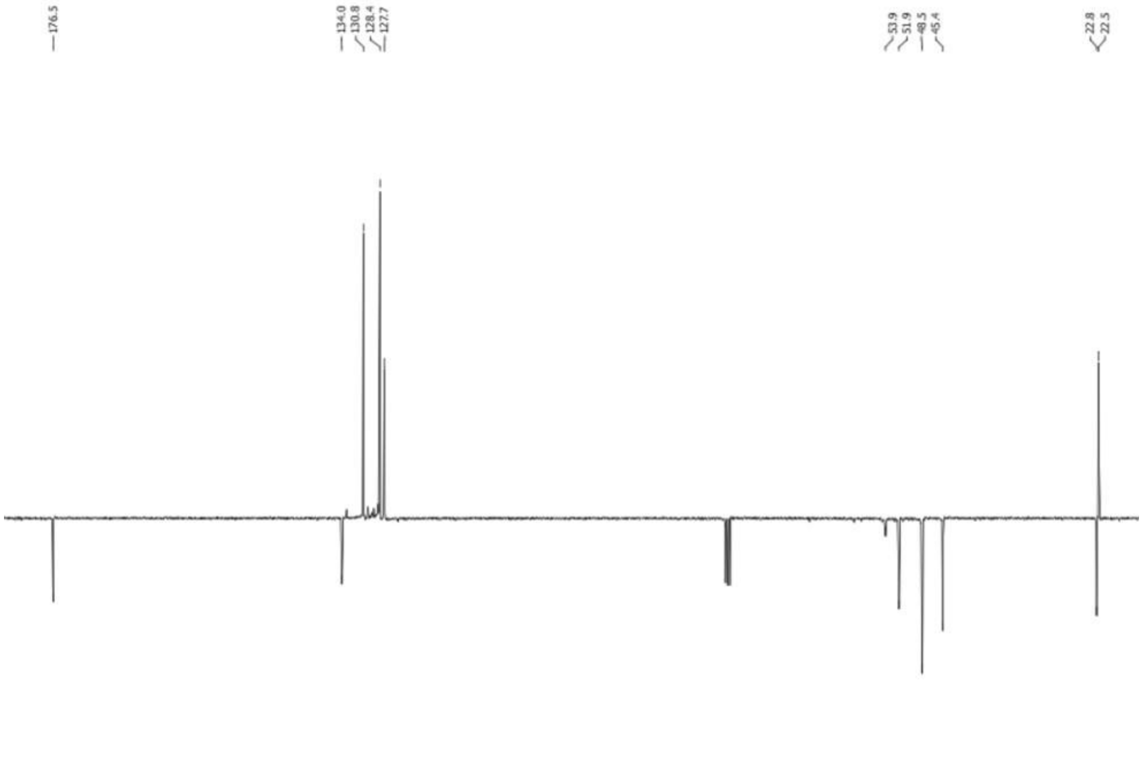

Figure S7B. $^{13}C\{^1H\}$ APT NMR spectrum of compound **9** in $CDCl_3$.

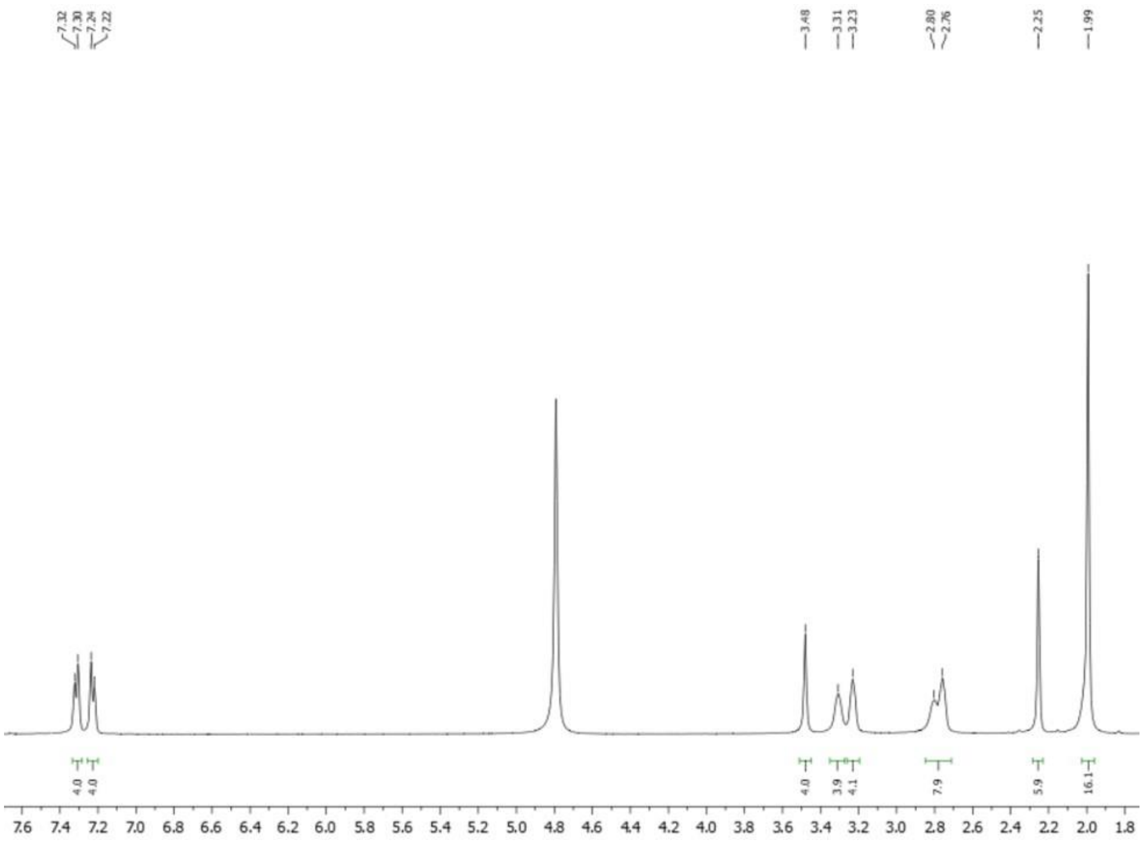

Figure S8A. ^{1}H NMR spectrum of compound **13** in D_2O.

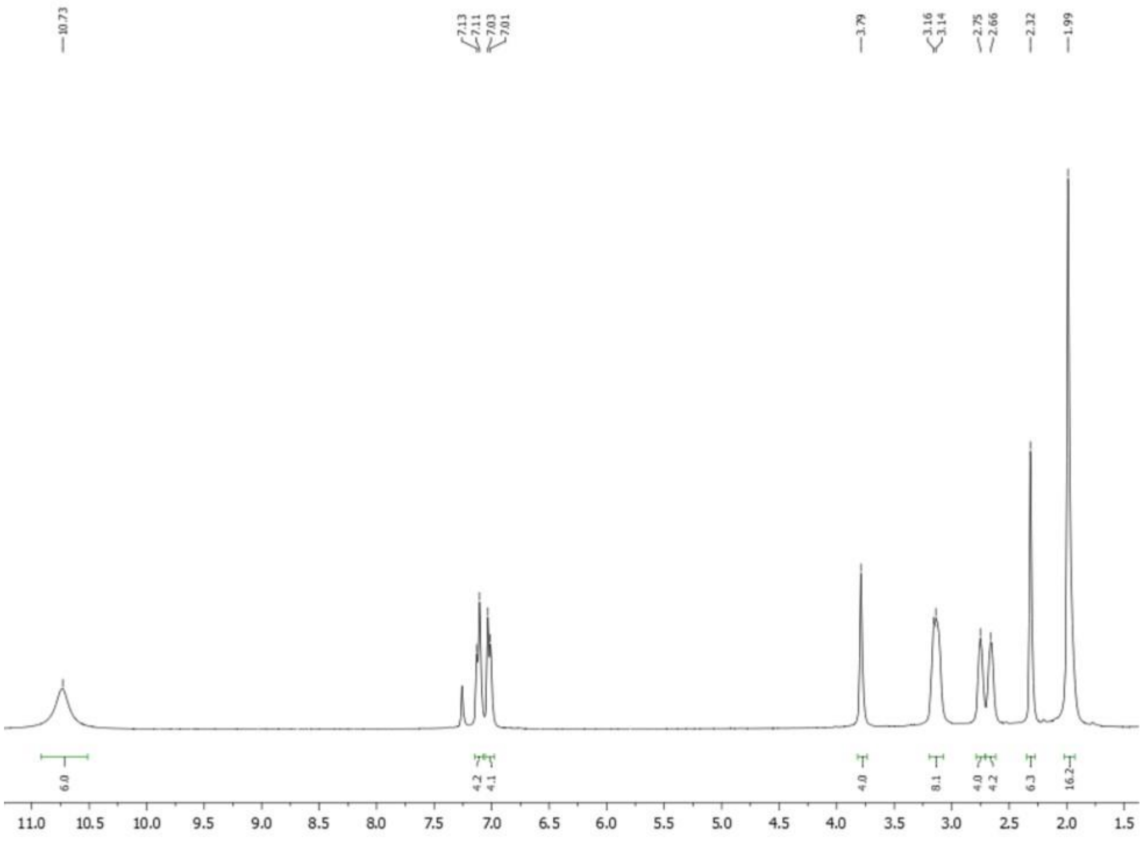

Figure S8B. ^{1}H NMR spectrum of compound **13** in $CDCl_3$.

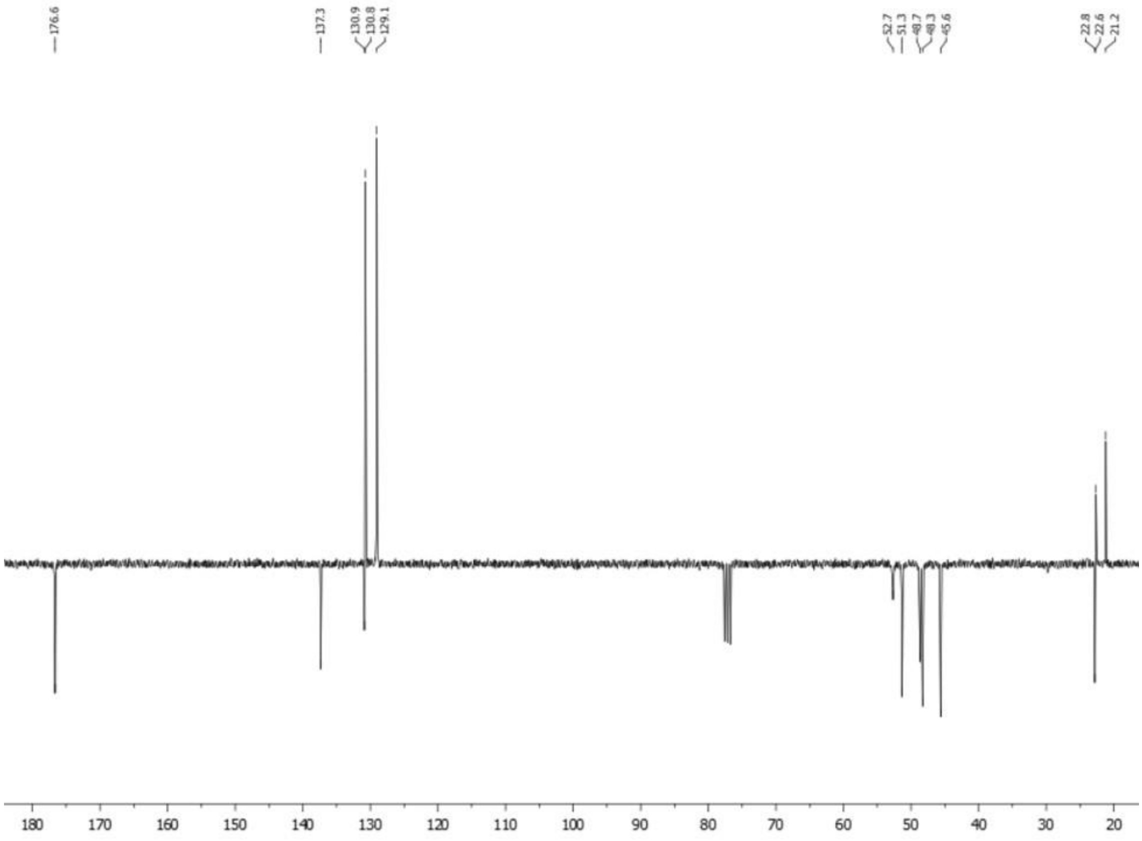

Figure S8C. $^{13}C\{^{1}H\}$ APT NMR spectrum of compound **13** in $CDCl_3$.

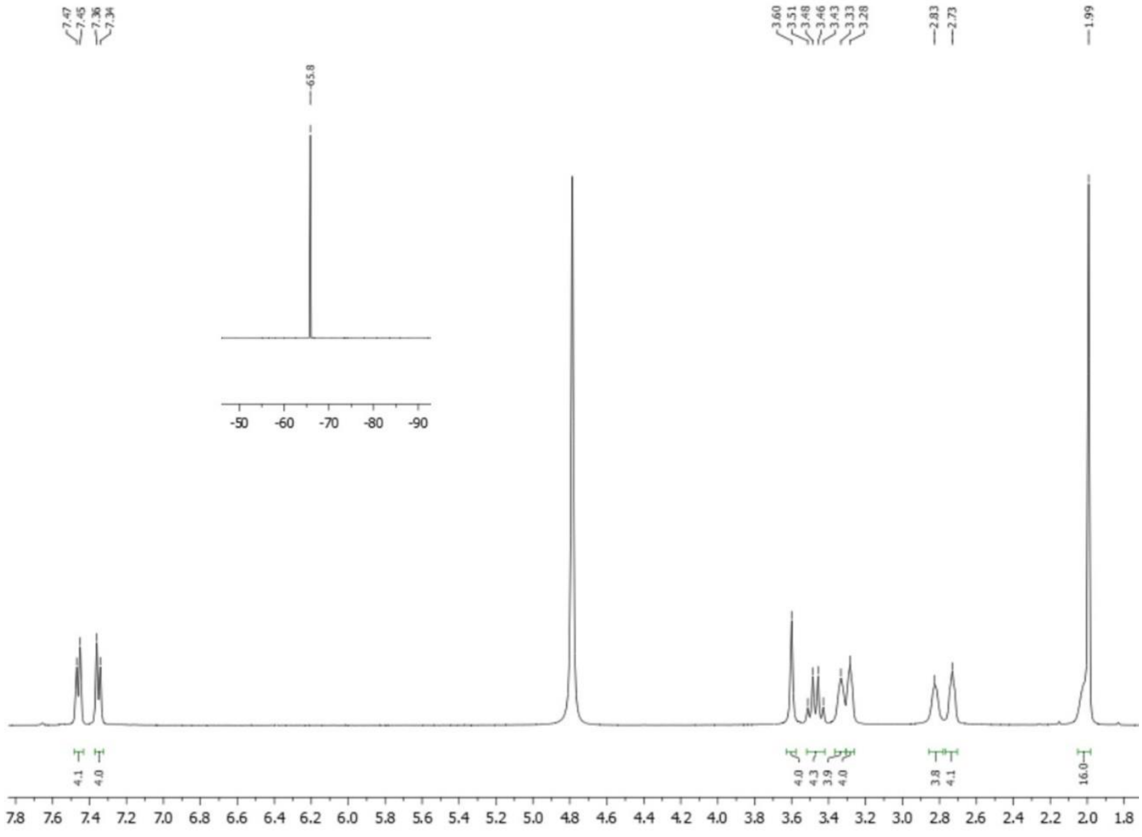

Figure S9A. ^{1}H and ^{19}F NMR spectra of compound **14** in D_2O.

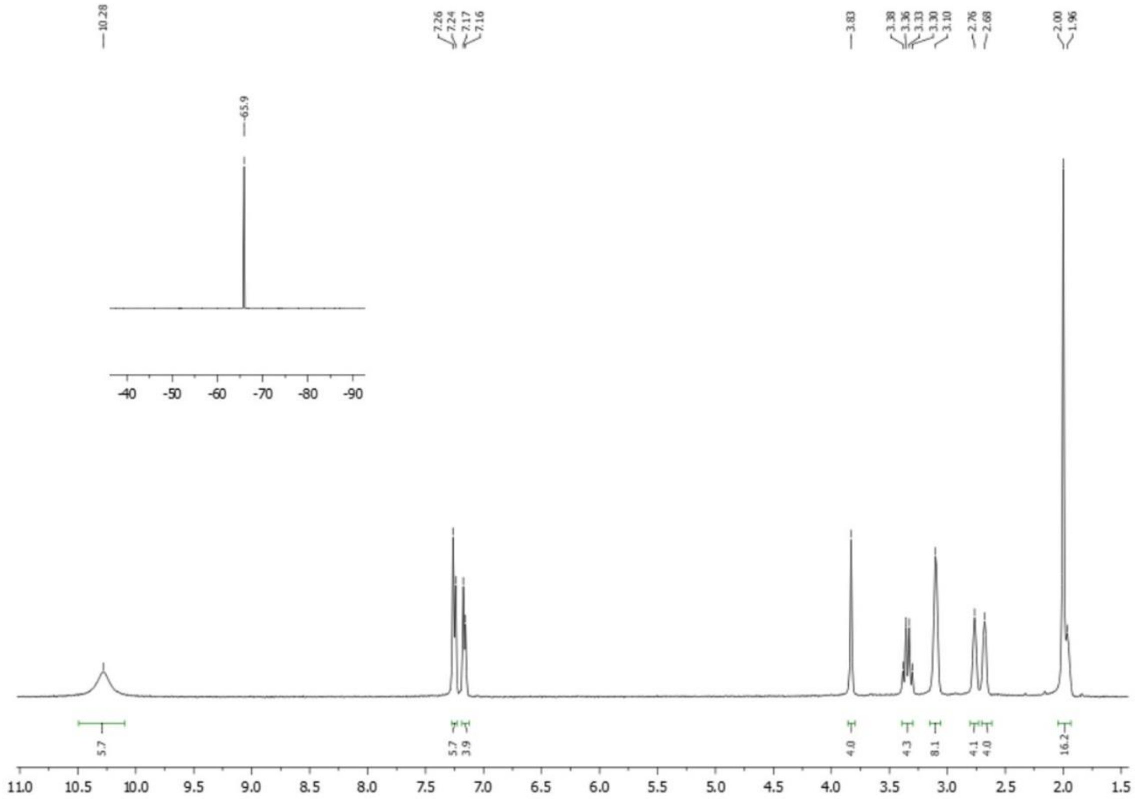

Figure S9B. ^{1}H and ^{19}F NMR spectra of compound **14** in $CDCl_3$.

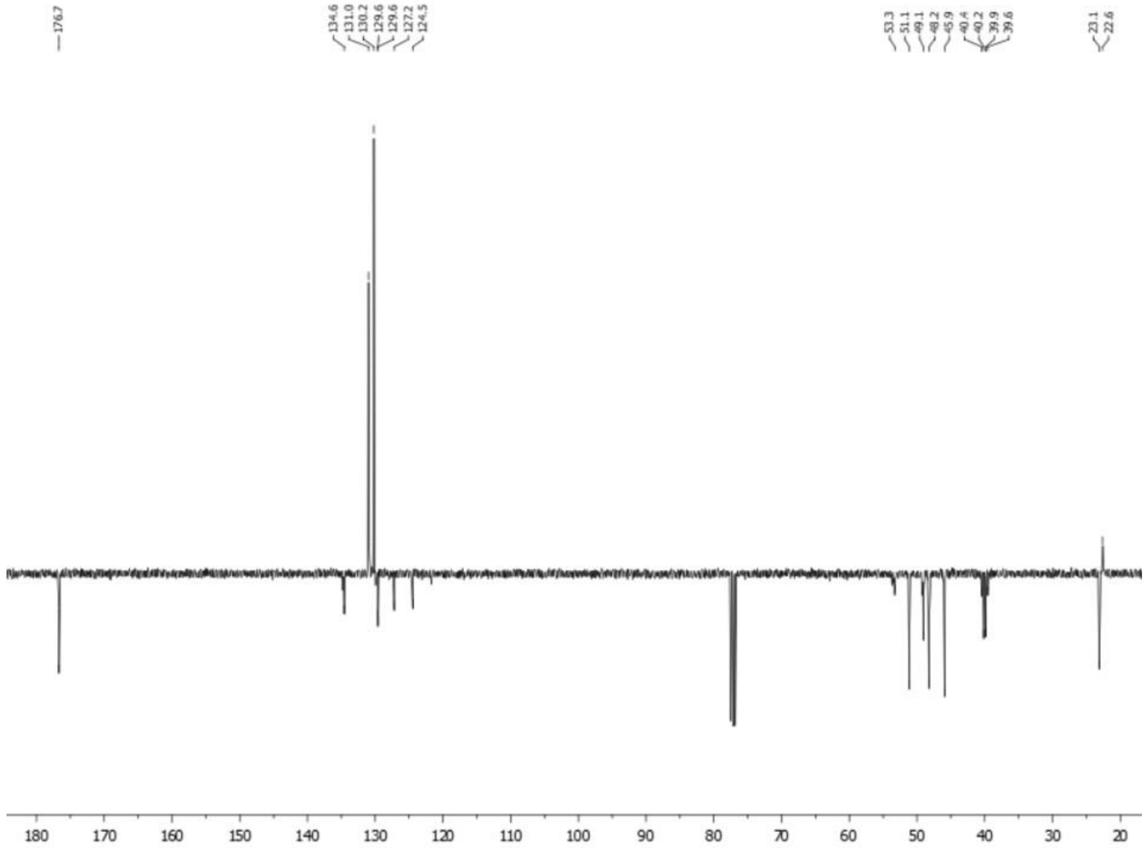

Figure S9C. $^{13}C\{^{1}H\}$ APT NMR spectrum of compound **9** in $CDCl_3$.

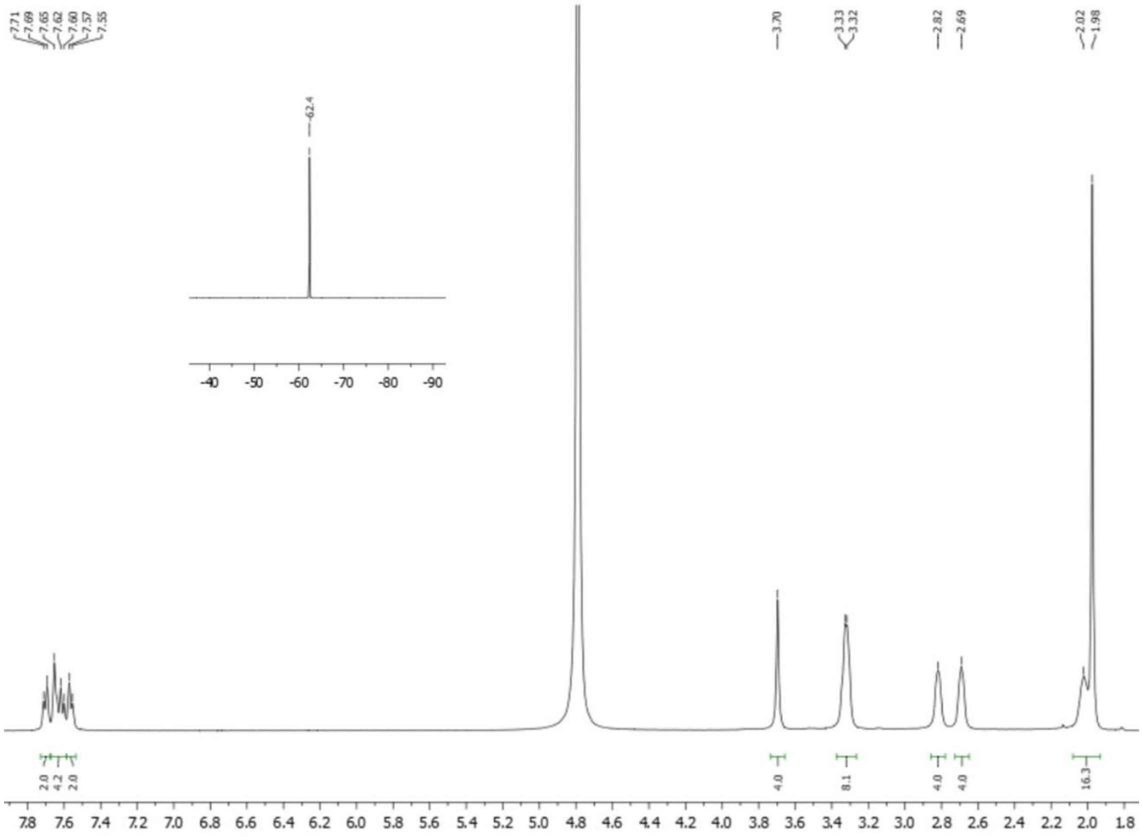

Figure S10A. ^{1}H and ^{19}F NMR spectra of compound **15** in D_2O.

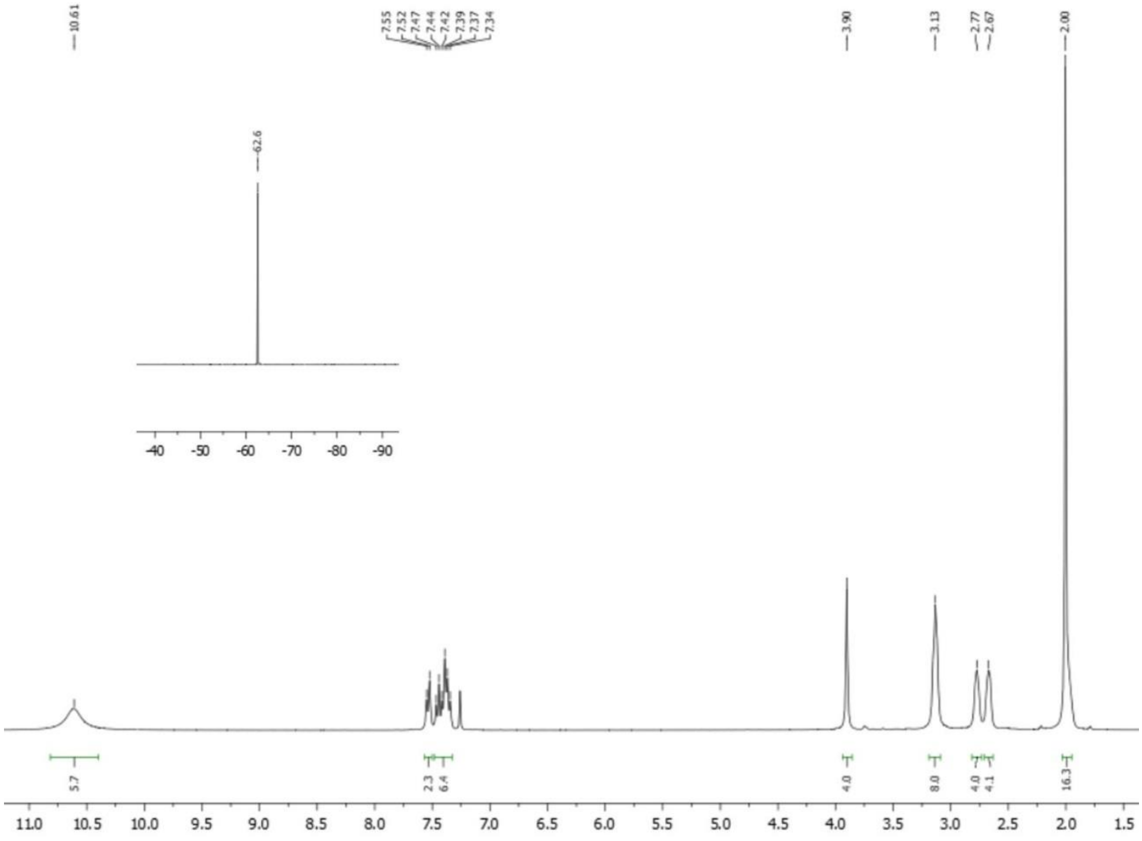

Figure S10B. ^{1}H and ^{19}F NMR spectra of compound **15** in $CDCl_3$.

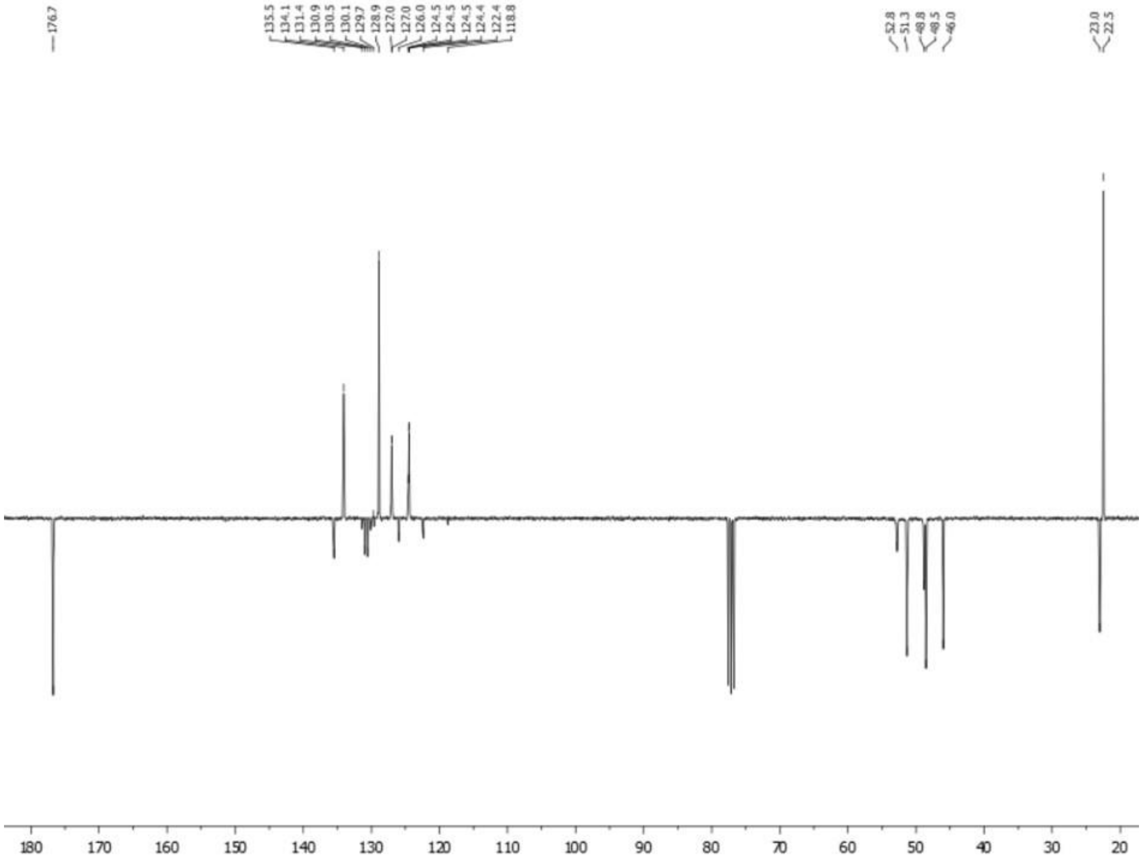

Figure S10C. $^{13}C\{^{1}H\}$ APT NMR spectrum of compound **15** in $CDCl_3$.

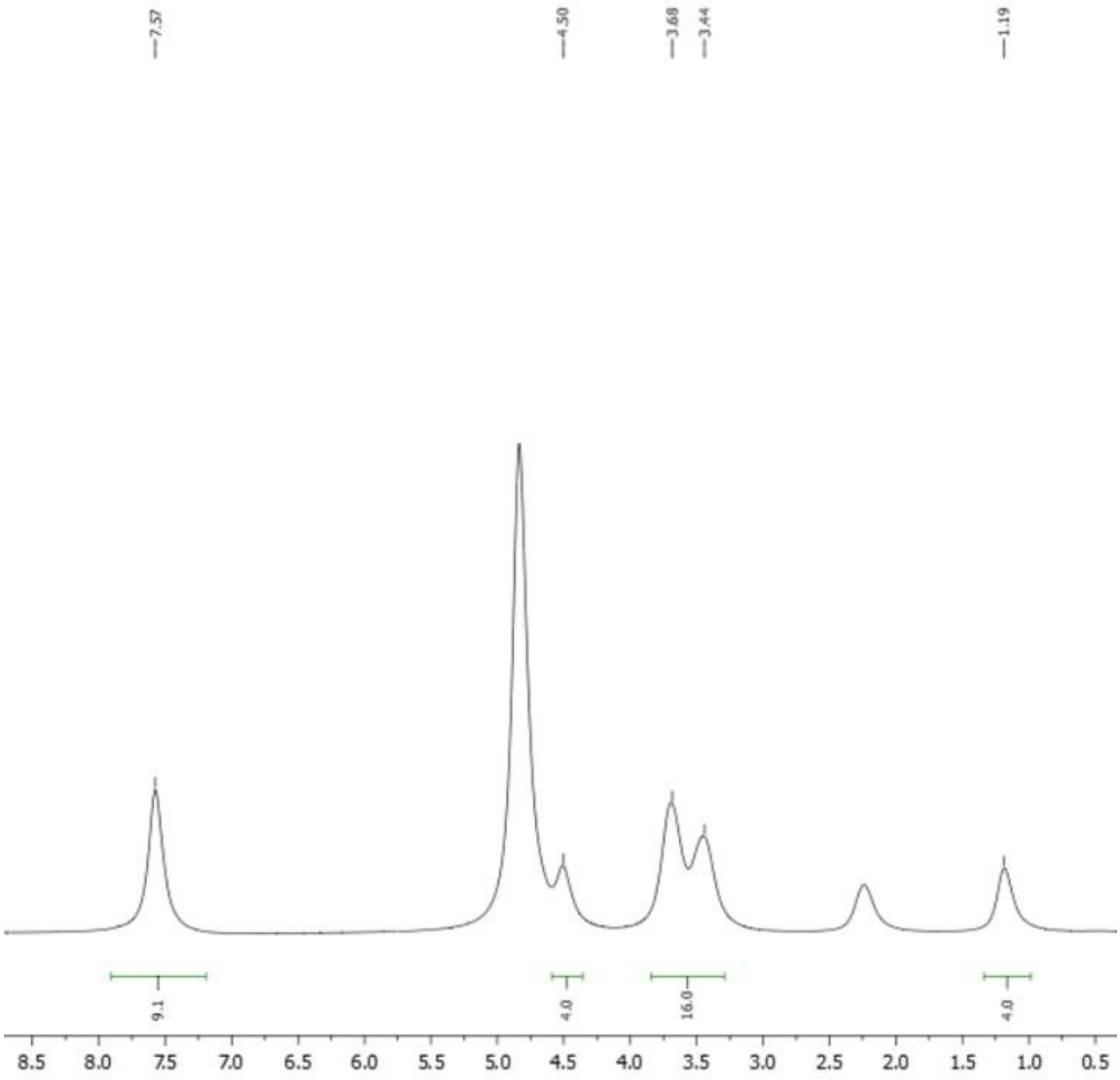

Figure S11A. ^1H NMR spectrum of compound **16** in $D_2O/(CD_3)_2CO$.

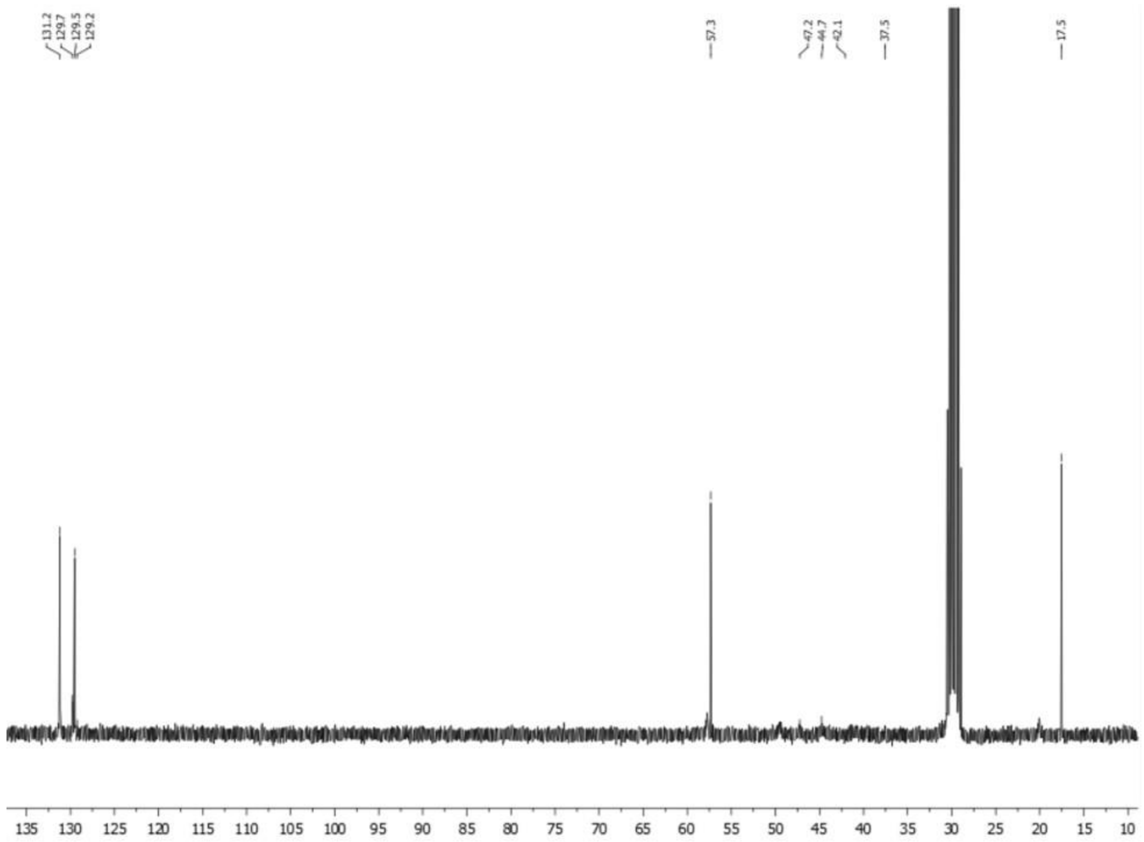

Figure S11B. $^{13}C\{^{1}H\}$ APT NMR spectrum of compound **16** in $D_2O/(CD_3)_2CO$.

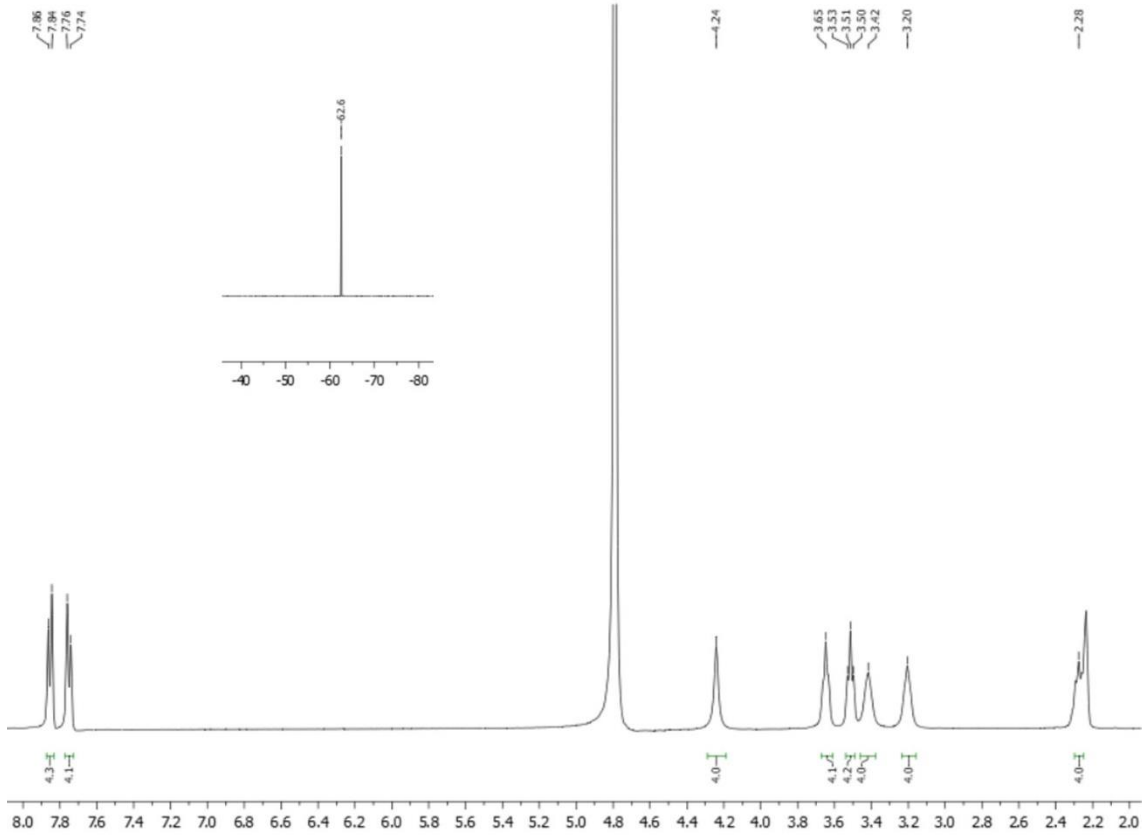

Figure S12A. ^{1}H and ^{19}F NMR spectra of compound **17** in $D_2O/(CD_3)_2CO$.

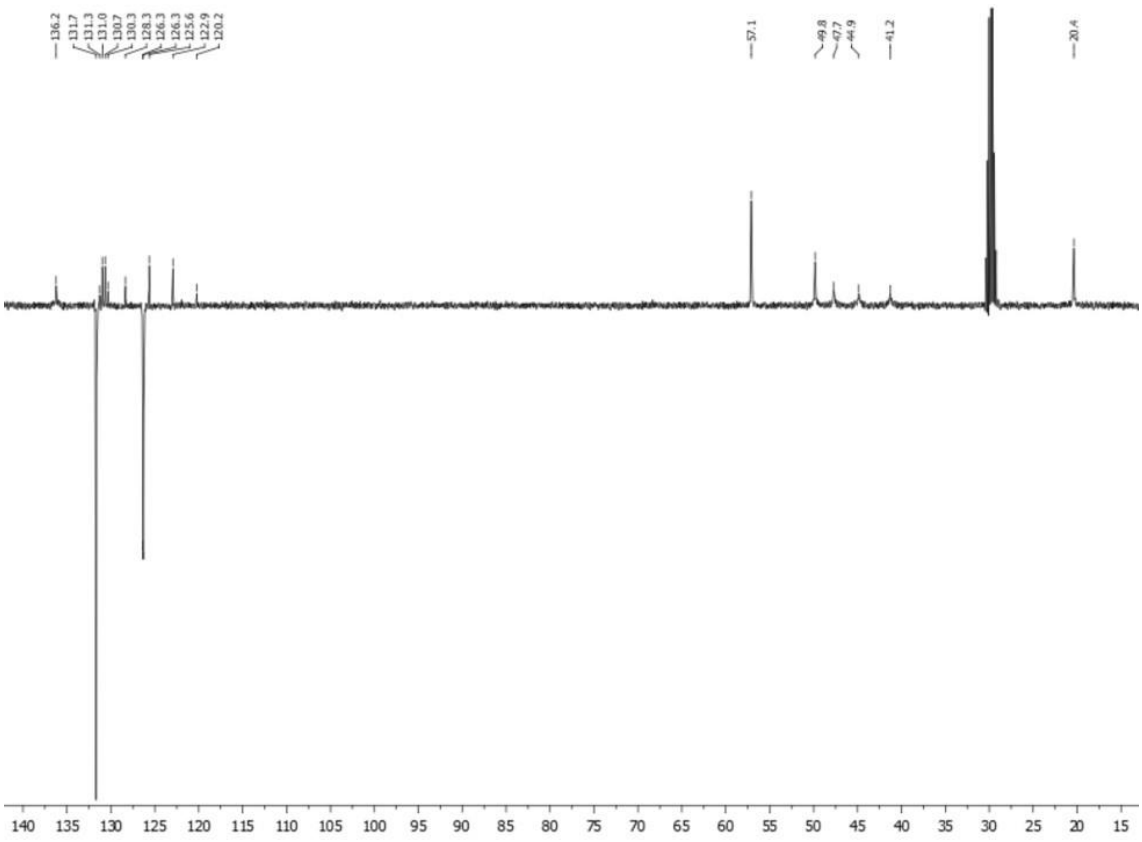

Figure S12B. $^{13}C\{^{1}H\}$ APT NMR spectrum of compound **17** in $D_2O/(CD_3)_2CO$.

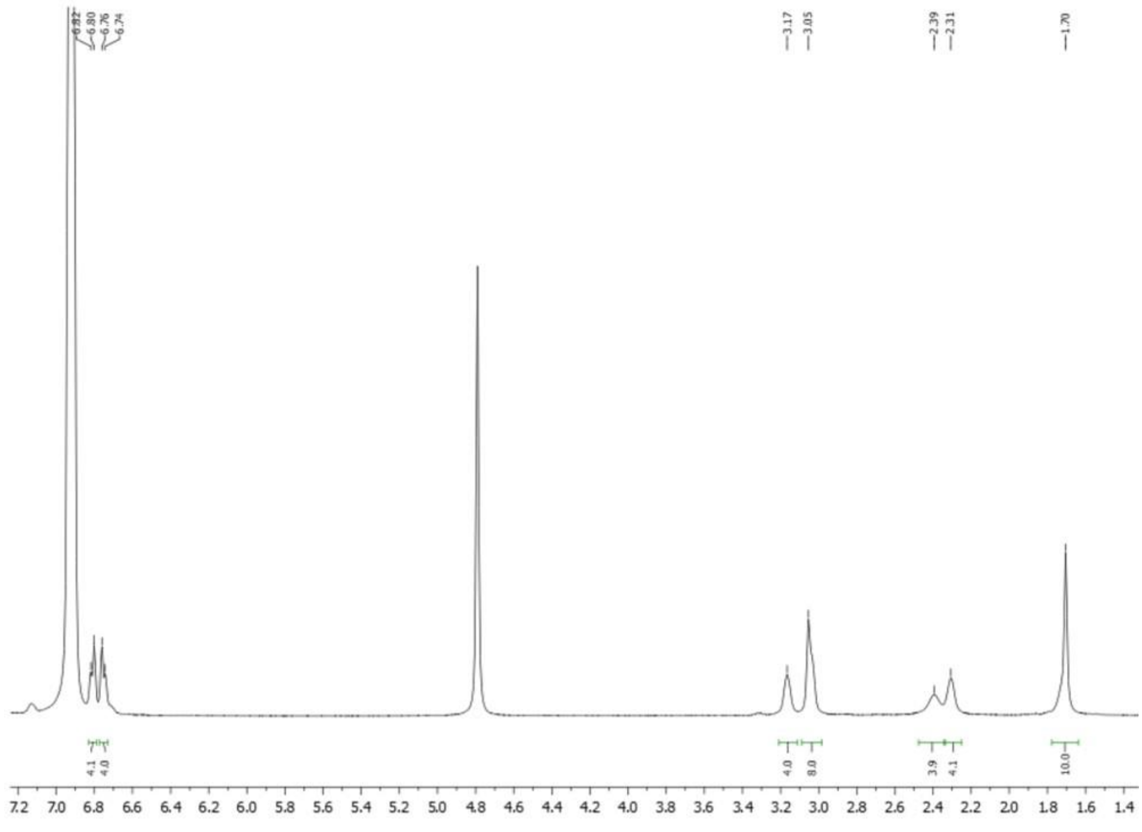

Figure S13A. ^{1}H spectrum of compound **18** in D_2O/C_6D_5N.

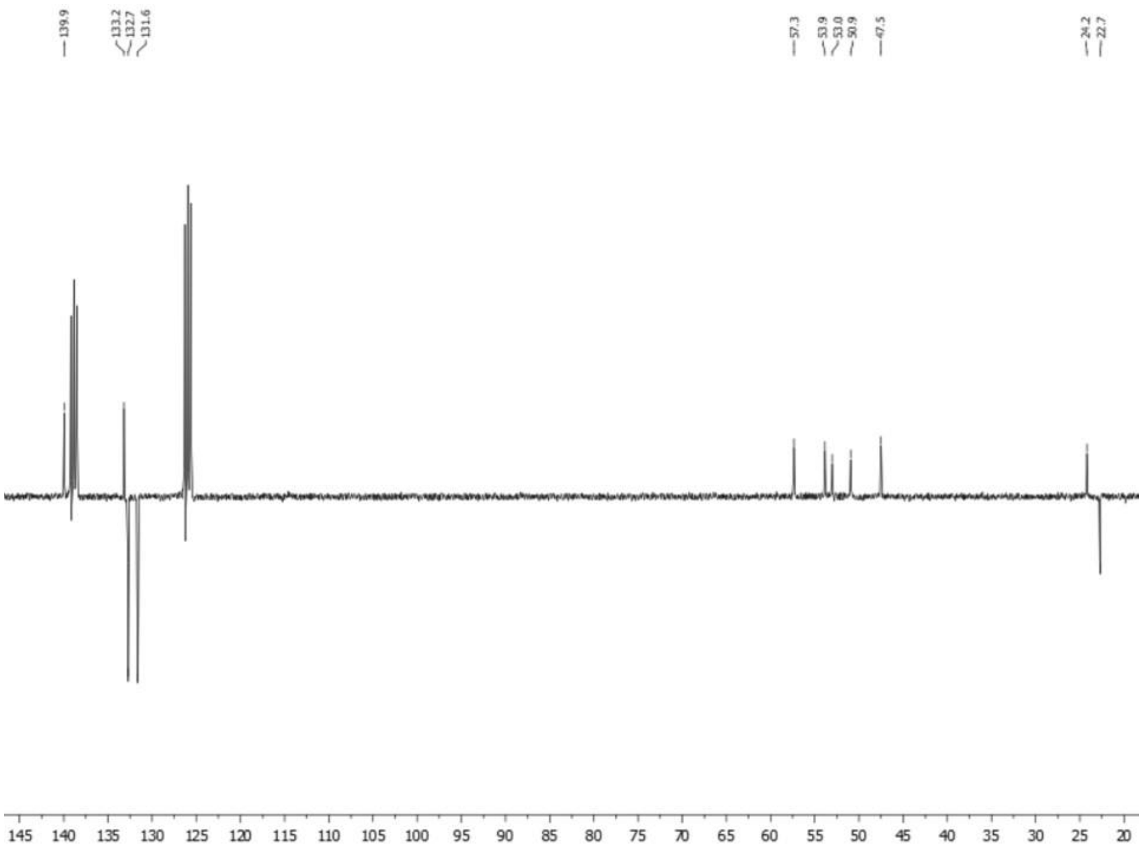

Figure S13B. $^{13}C\{^{1}H\}$ APT NMR spectrum of compound **18** in D_2O/C_6D_5N.

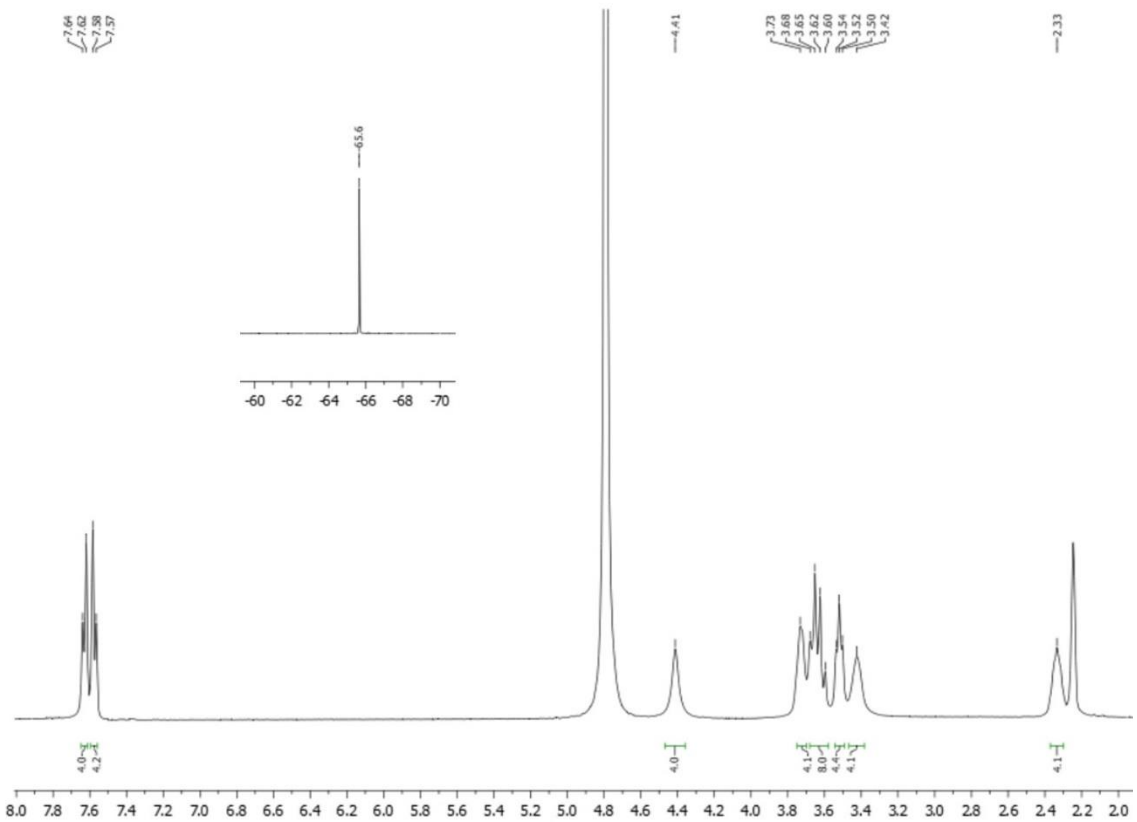

Figure S14A. ^{1}H and ^{19}F NMR spectra of compound **19** in $D_2O/(CD_3)_2CO$.

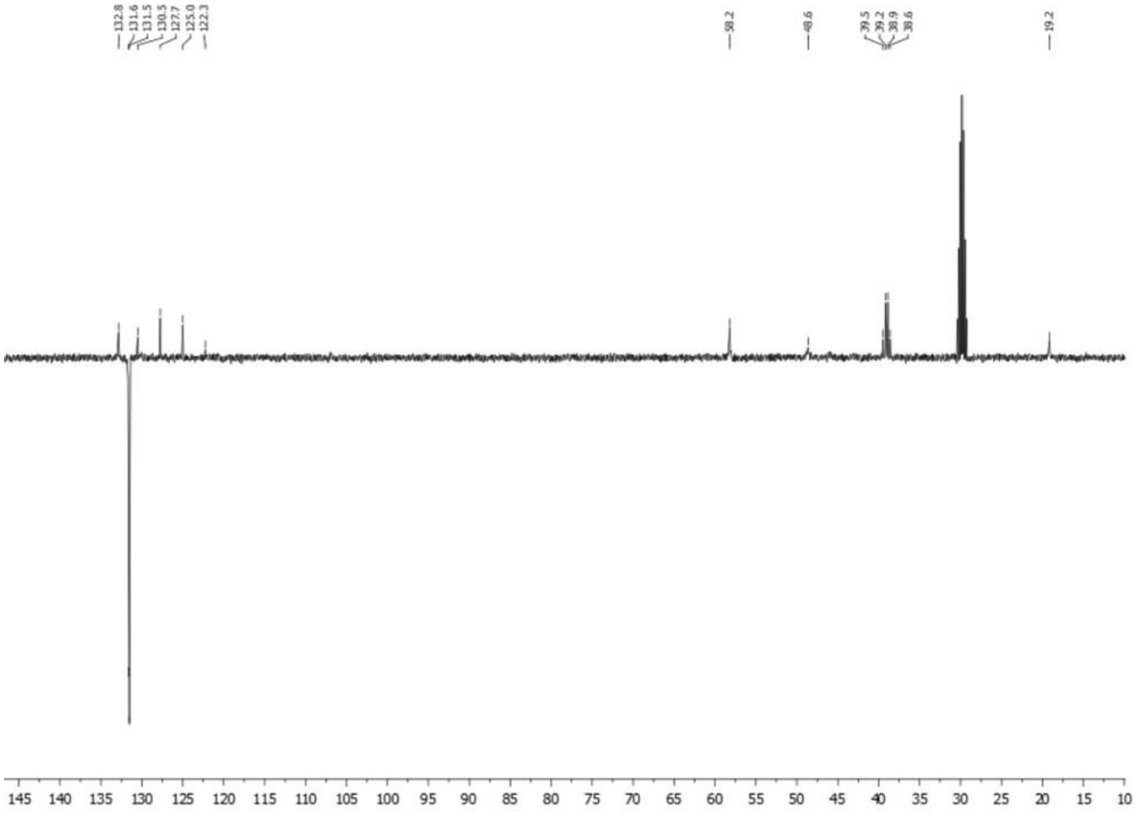

Figure S14B. $^{13}C\{^1H\}$ APT NMR spectrum of compound **19** in $D_2O/(CD_3)_2CO$.

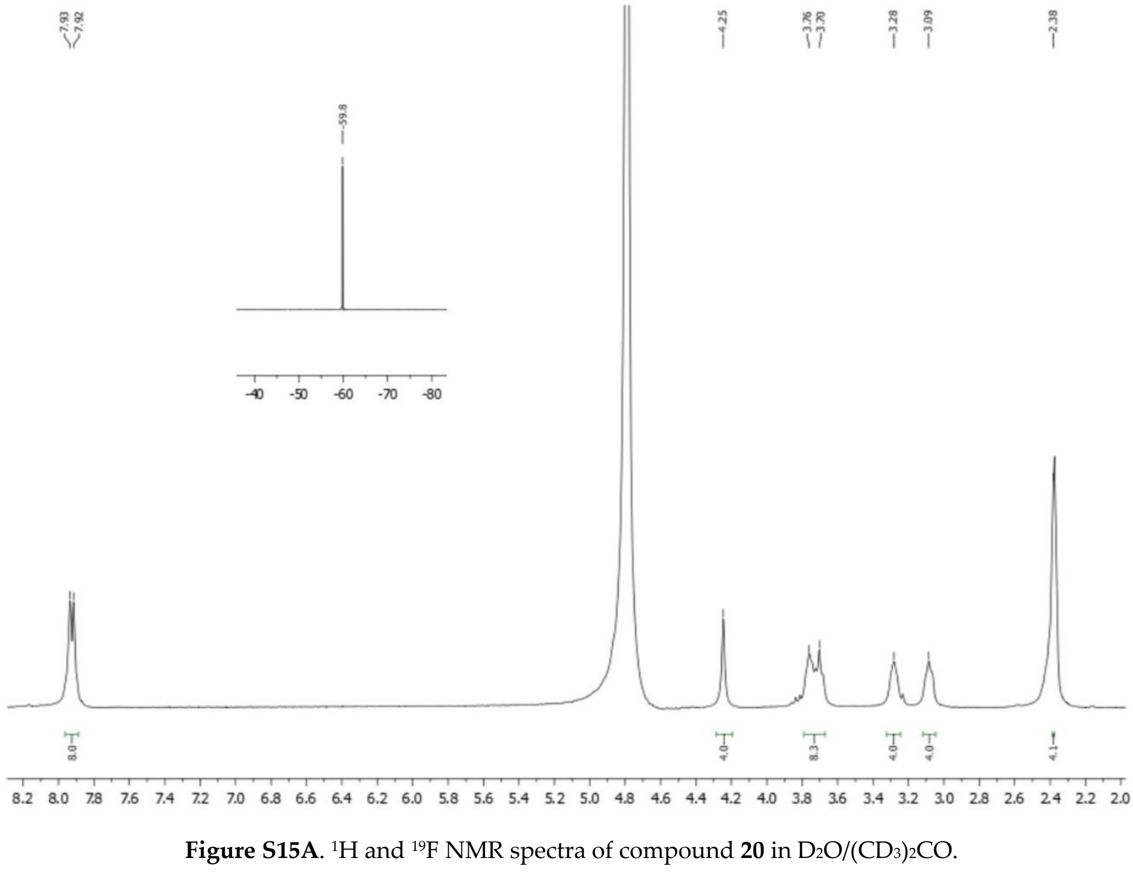

Figure S15A. ^1H and ^{19}F NMR spectra of compound **20** in $D_2O/(CD_3)_2CO$.

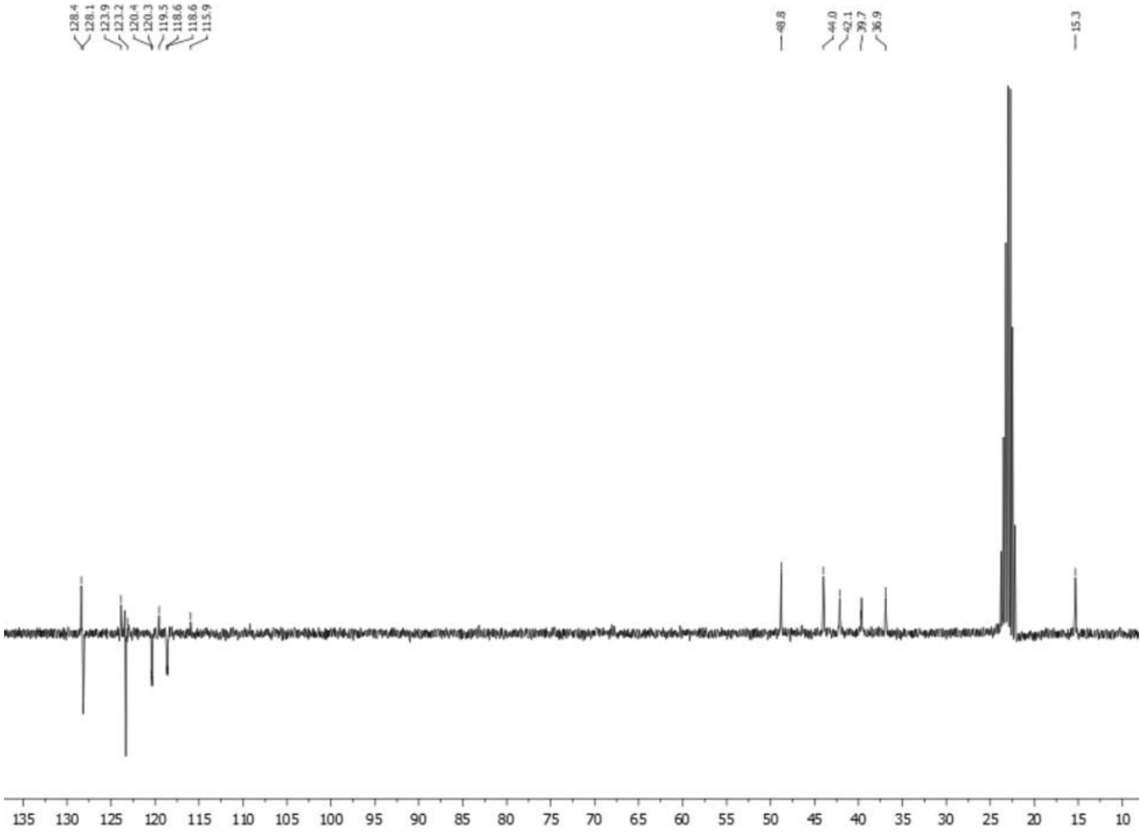

Figure S15B. $^{13}C\{^{1}H\}$ APT NMR spectrum of compound **20** in $D_2O/(CD_3)_2CO$.

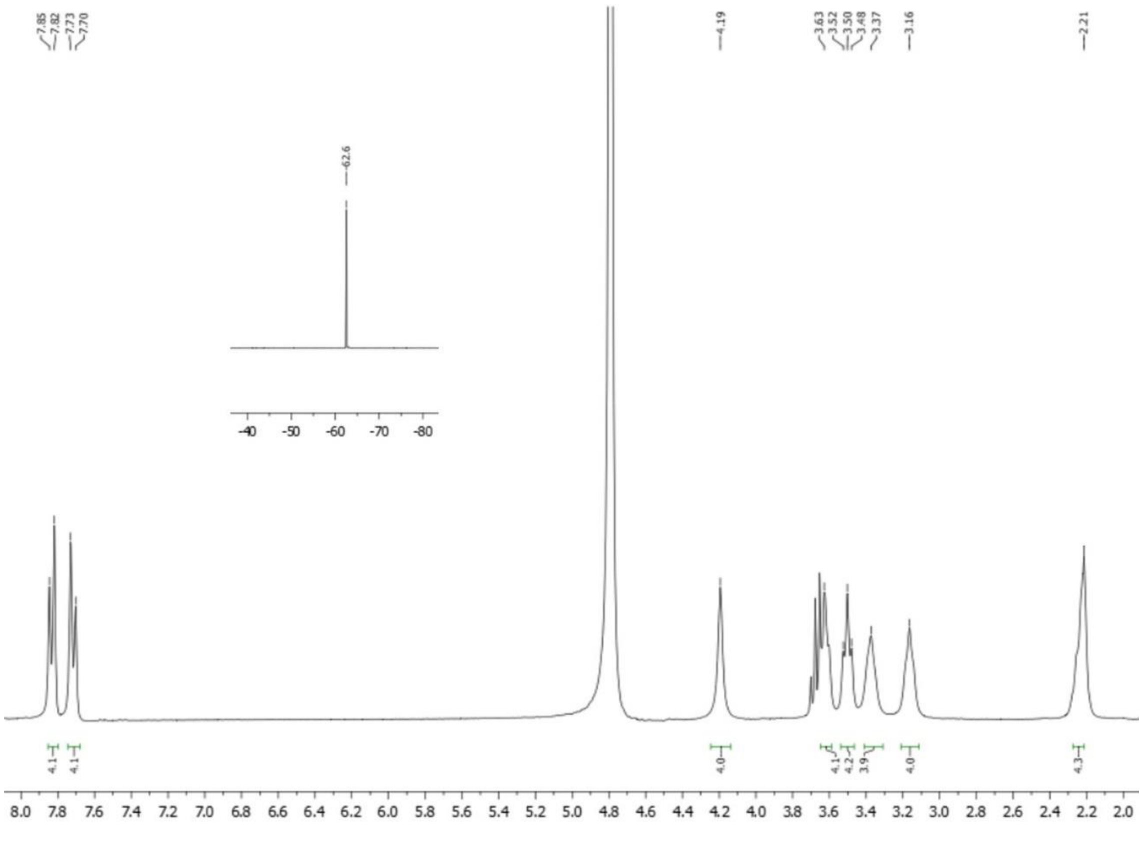

Figure S16A. ^{1}H and ^{19}F NMR spectra of compound **21** in $D_2O/(CD_3)_2CO$.

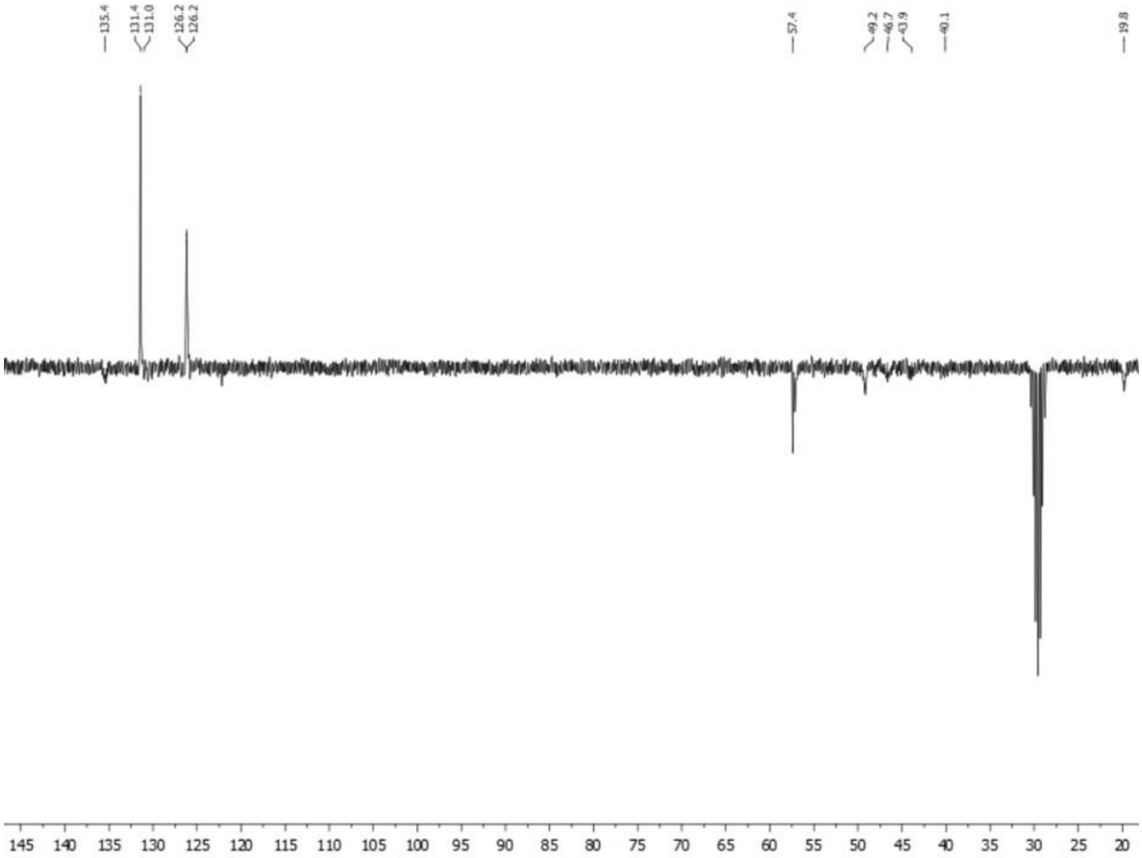

Figure S16B. $^{13}C\{^{1}H\}$ APT NMR spectrum of compound **21** in $D_2O/(CD_3)_2CO$.

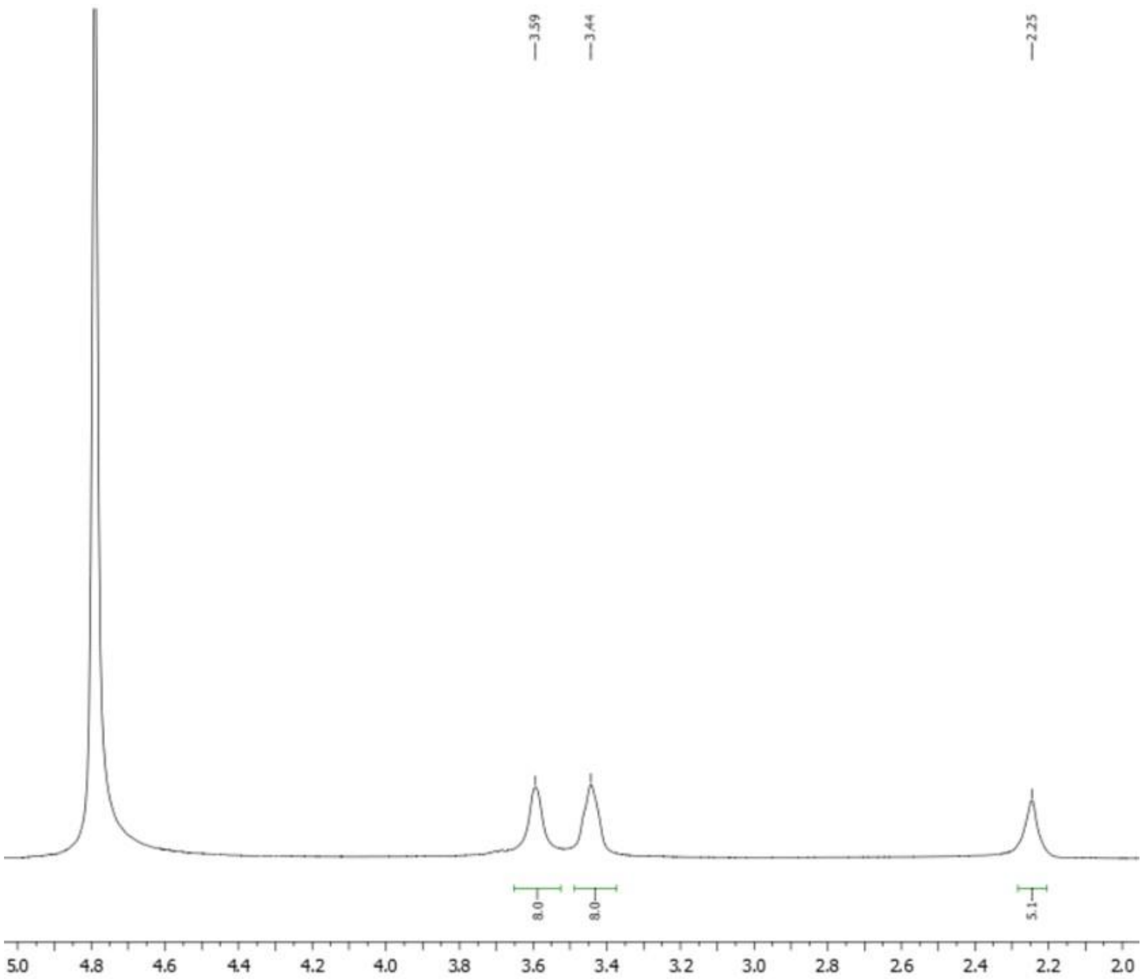

Figure S17A. ^{1}H NMR spectrum of compound **22** in $D_2O/(CD_3)_2CO$.

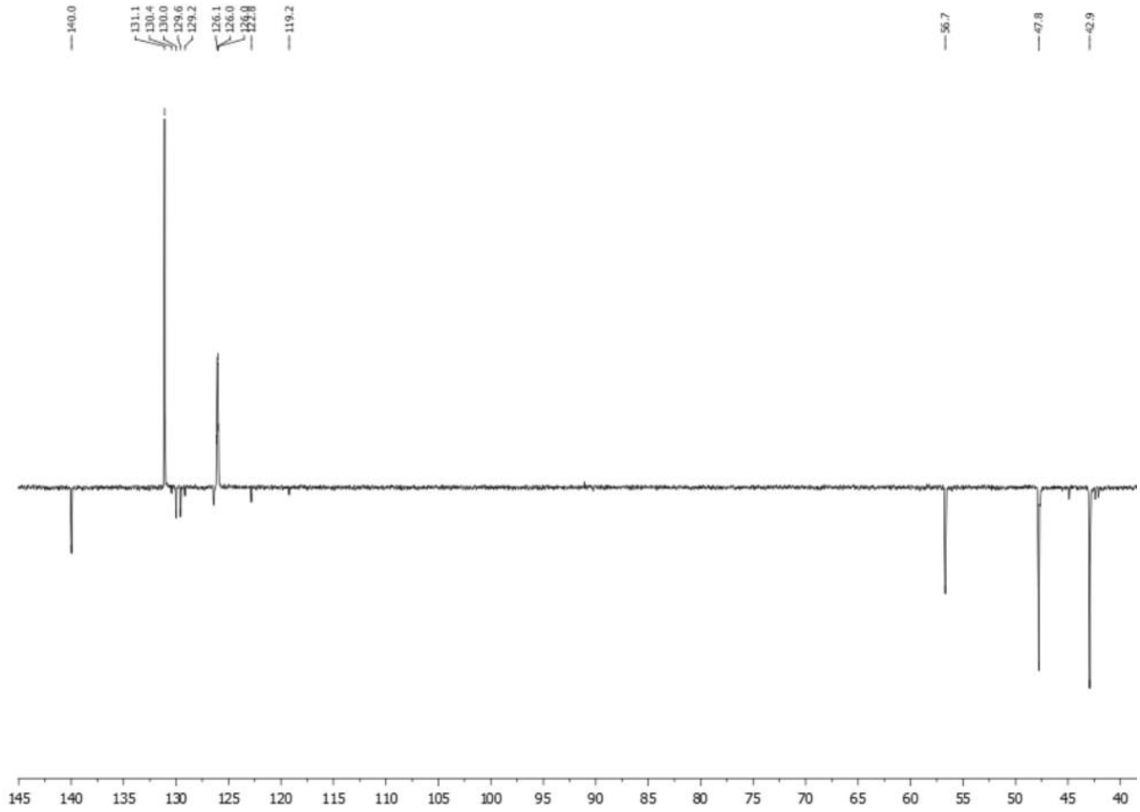

Figure S17B. $^{13}C\{^{1}H\}$ APT NMR spectrum of compound **22** in $D_2O/(CD_3)_2CO$.

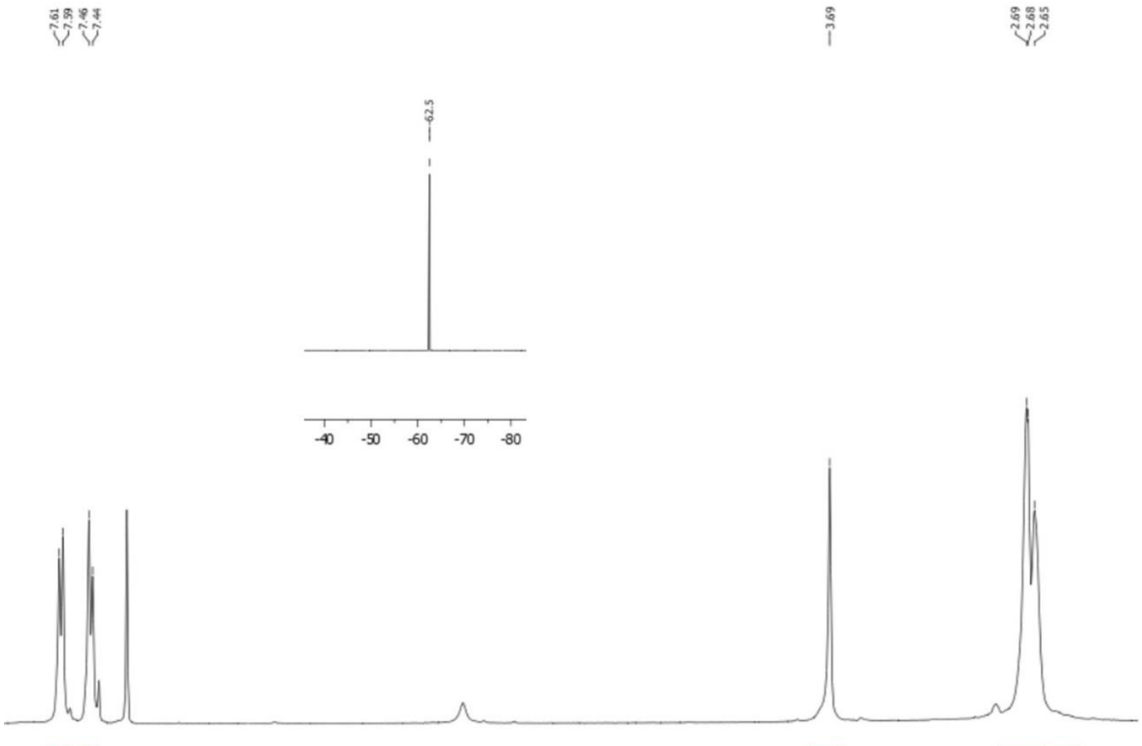

Figure S18A. ^{1}H and ^{19}F NMR spectra of compound **24** in $CDCl_3$.

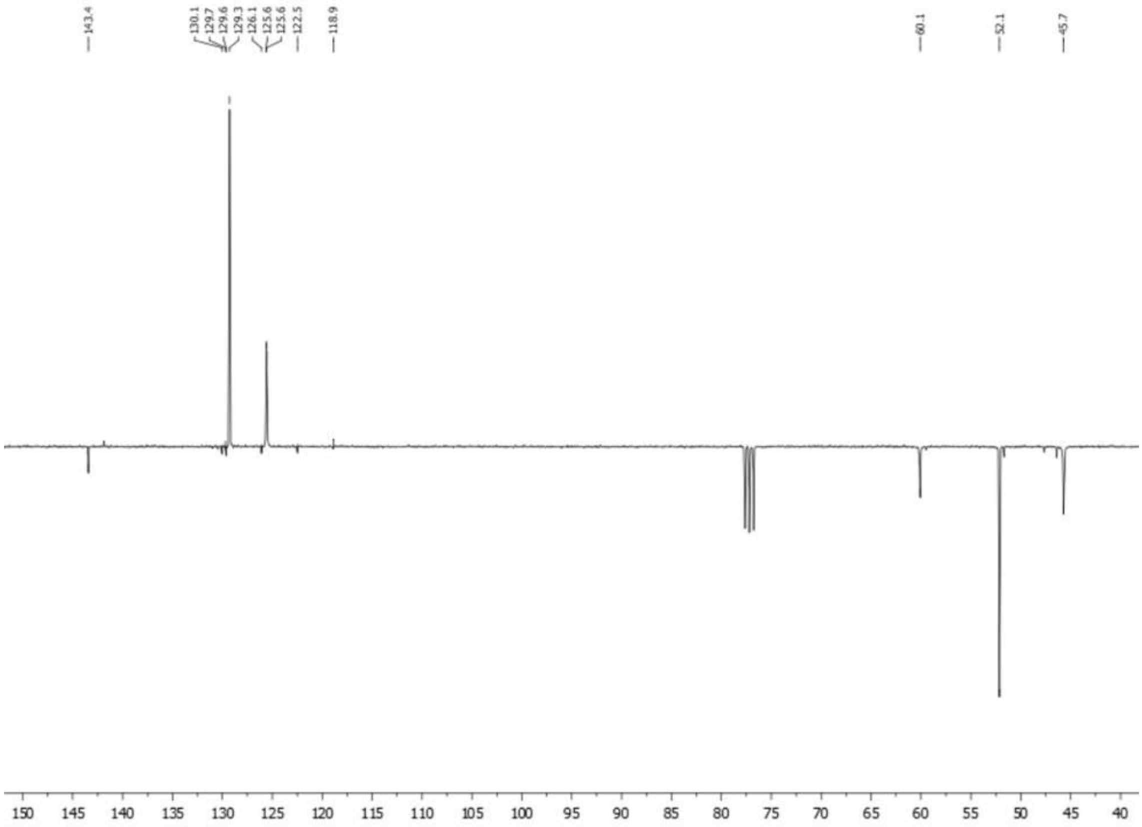

Figure S18B. $^{13}C\{^{1}H\}$ APT NMR spectrum of compound **24** in $CDCl_3$.

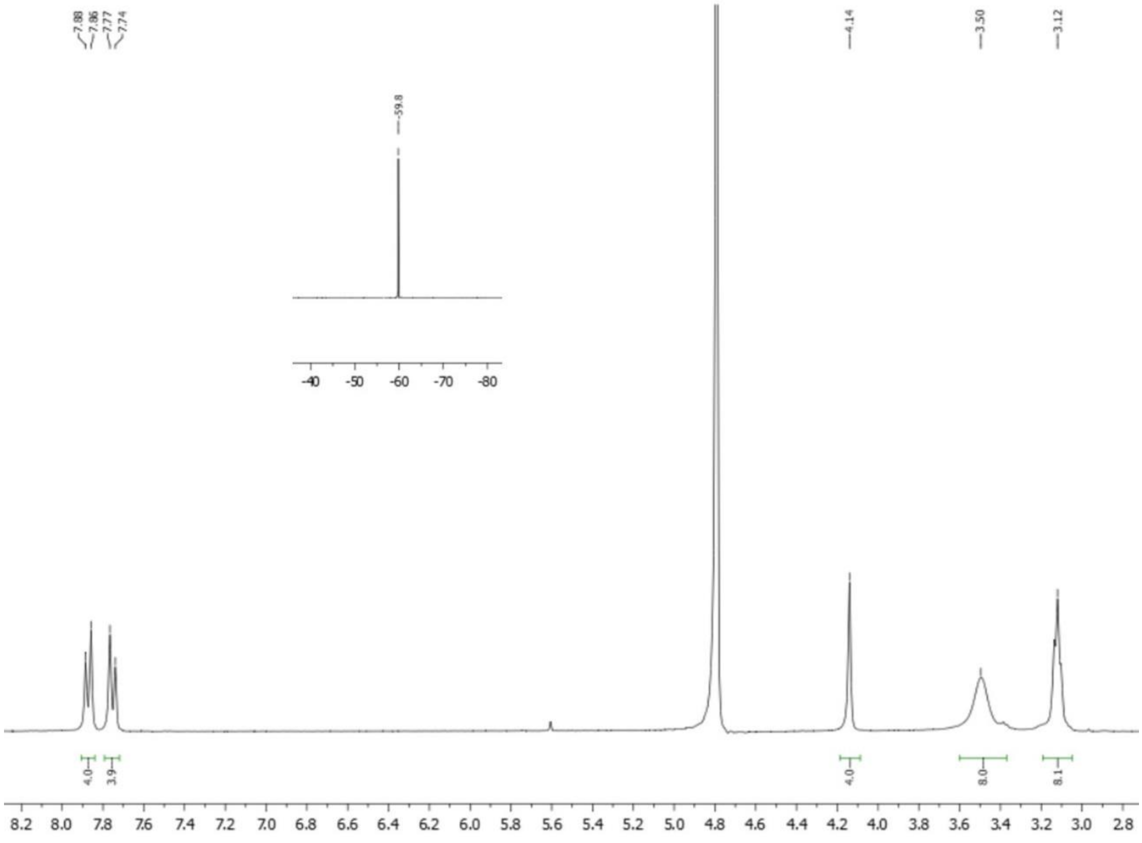

Figure S19A. ^{1}H and ^{19}F NMR spectra of compound **25** in $D_2O/(CD_3)_2CO$.

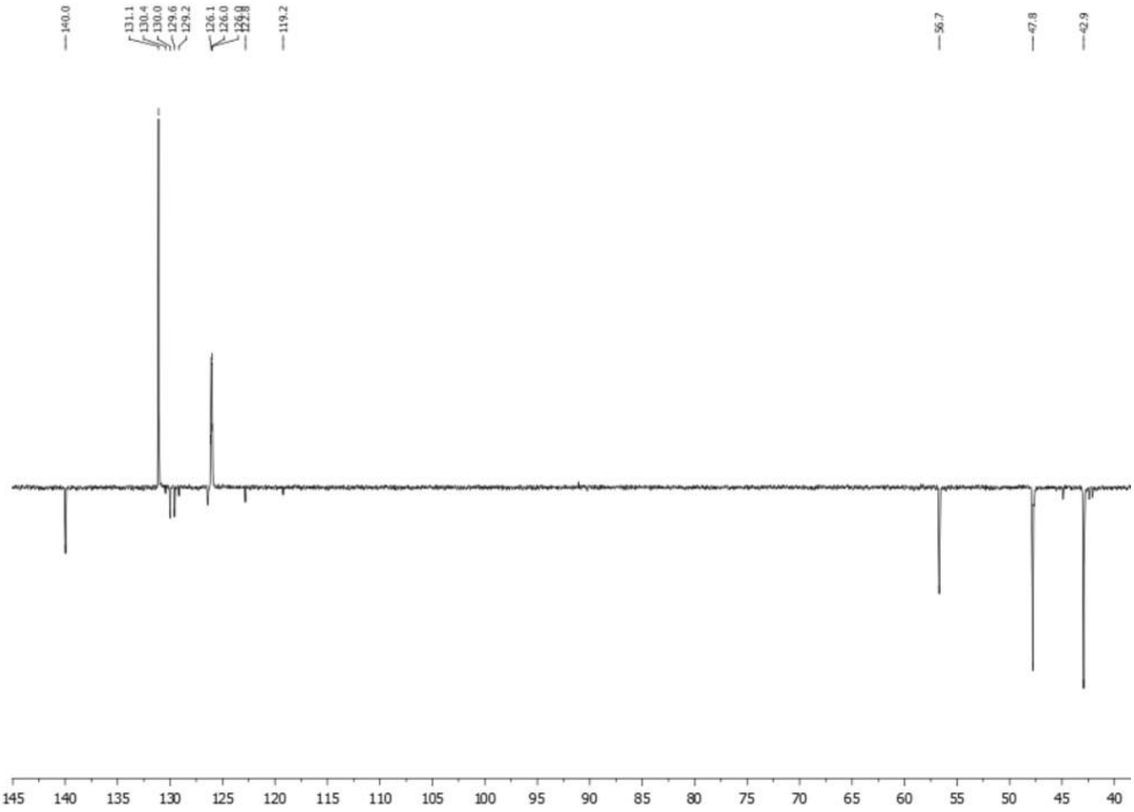

Figure S19B. $^{13}C\{^{1}H\}$ APT NMR spectrum of compound **25** in $D_2O/(CD_3)_2CO$.
